# Spatial Stochastic Intracellular Kinetics: A Review of Modelling Approaches

**DOI:** 10.1007/s11538-018-0443-1

**Published:** 2018-05-21

**Authors:** Stephen Smith, Ramon Grima

**Affiliations:** School of Biological Sciences, University of Edinburgh, Mayfield Road, Edinburgh, EH9 3JR Scotland, UK

**Keywords:** Reaction–diffusion master equation, Spatial models, Brownian dynamics

## Abstract

Models of chemical kinetics that incorporate both stochasticity and diffusion are an increasingly common tool for studying biology. The variety of competing models is vast, but two stand out by virtue of their popularity: the reaction–diffusion master equation and Brownian dynamics. In this review, we critically address a number of open questions surrounding these models: How can they be justified physically? How do they relate to each other? How do they fit into the wider landscape of chemical models, ranging from the rate equations to molecular dynamics? This review assumes no prior knowledge of modelling chemical kinetics and should be accessible to a wide range of readers.

## Introduction

Chemical reactions are the building blocks of biology. Substrates bind to enzymes, messenger RNA binds to ribosomes, proteins bind to DNA, and amino acids bind to each other—the cumulative effect is ultimately life as we know it. It is perhaps unintuitive to think in these terms, but any observable behaviour of a living organism (e.g. a human reading this sentence) can be understood as a series of interactions between molecules. The question of how microscopic chemistry gives rise to macroscopic biology is one of the greatest open scientific problems. Modelling is key to answering this question: our understanding of the underlying chemistry is good, as is our knowledge of the observable biology; what is missing is a model linking the distinct scales together.

A system can be modelled in a great variety of ways, but not all models are equally useful. It is theoretically possible to model the biochemistry in an entire cell using Schrödinger’s equation, but it would not be appropriate. The challenge is to select a model incorporating the salient details of a system while leaving out the extraneous ones.

For instance, consider a simple molecule like carbon dioxide, CO$_2$. At the coarsest level, we could model the *concentration* of CO$_2$, which refers to the total number of carbon dioxide molecules divided by the volume of whatever container the molecules are in (such as a cell or a test tube). The numerical value which we assign to the concentration will depend on the concentrations of other chemical species in the container and the rates of any reactions which involve these species. For instance, there might be some molecules of carbon monoxide, CO, and some molecules of oxygen, O, and there might be a reaction of the form:

1 $$\begin{aligned} \hbox {CO}+\hbox {O}\xrightarrow {k}\hbox {CO}_2, \end{aligned}$$

where *k* denotes the rate at which CO and O are converted into CO$_2$. The basic model which describes chemical kinetics at this level of complexity is the rate equations (REs) (Chen et al. 2010).

Alternatively, at a considerably more complex level we could model each atom of each molecule individually, by considering the forces exerted by each atom in the volume on every other atom. The three atoms which make up any single CO$_2$ molecule are bonded together and so will exert very strong forces on each other (these forces are traditionally modelled as springs), but they will also experience weaker forces from the rest of the atoms (e.g. electrostatic forces). This is a more complex model because if our container contains, say, 1000 C atoms and 2000 O atoms we will need to keep track of 9000 distinct quantities (the locations of each atom in 3 spatial dimensions) rather than 3 in the REs (the concentrations of each species). The basic model which describes chemical kinetics at this level of complexity is molecular dynamics (MD) (Frenkel 2001).

**Fig. 1:**
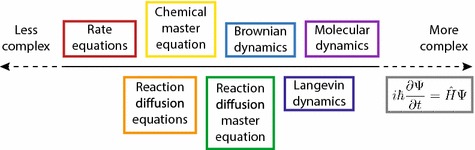
The scale of model complexity, ranging from coarse to extremely complex. Between the extremes are the models of interest in chemical kinetics, ranging from the rate equations (red) to molecular dynamics (violet). Reviews of the models *not* studied in depth in this article can be found in Refs. Murray (2001), Gillespie et al. (2013) and Durrant (2011)

There are a huge variety of models spanning the range of complexity from the REs to MD. We can imagine these models as points on a “complexity scale”, ranging from the coarsest (the REs) to the extremely complex (the Schrödinger equation), as shown in Fig. 1. On the left, we find the REs. As we move up the complexity scale, we gain microscopic detail at the cost of more difficult mathematics or longer computation times, ultimately resulting in MD. For example, one of the main assumptions behind the REs is that the diffusion coefficients of all molecules are infinitely fast—relaxing this assumption pushes us up the complexity scale to a model known as the reaction–diffusion equations (RDEs) (Murray 2001), which is always going to be more accurate than the REs, but correspondingly will always incur a greater computational cost.

The two models which we focus on in this review are at an intermediate level of complexity: the reaction–diffusion master equation (RDME) (Gillespie et al. 2014) and Brownian dynamics (BD) (Lipkov et al. 2011). As we will go on to show, these models are very different, though they both model the same kinds of processes at roughly the same level of detail. Unlike the REs, the RDME and BD both acknowledge that molecules are discrete entities, but they do not go down to atomic-level resolution like MD. The RDME and BD do not explicitly model water molecules (unlike MD), but they do model the *effect* of water molecules (diffusion) which is beyond the scope of the REs.

The reason for focussing on the RDME and BD is that they are likely to be the appropriate level of modelling detail for understanding the link between microscopic chemistry and macroscopic biology (Klann and Koeppl 2012). They are the simplest models which explicitly model individual reactions and the diffusion of individual molecules, and so are not too distant from chemistry as we understand it intuitively. Yet they are not too computationally intensive: both models have already been used to study systems on the scale of an entire cell (Fange and Elf 2006; Sturrock et al. 2013; Lipkow et al. 2005; Andrews et al. 2010), and it is surely only a matter of time until multicellular organisms are within their scope.

It is worth mentioning at this point that this review will not go into detail about the computational methods used to implement these models in practice (the so-called simulation algorithms). A relatively recent and comprehensive review of this issue can be found in Ref. Erban et al. (2007). Instead, our concern will be with the validity of the RDME and BD, how they are related, when it is appropriate to use them, and how they fit into the modelling complexity scale. Atypically, the subject of our studies will be the models themselves rather than the systems to which they could be applied.

Our review is split into three sections. In Sect. 2 we discuss the mathematical origins of the RDME, followed by a detailed analysis of how it relates to other models on the complexity scale. In Sect. 3 we discuss the physical origins of BD, followed by a rigorous discussion of its position in the complexity scale. In Sect. 4 we briefly discuss the extent to which BD and the RDME agree with each other. We conclude with a discussion in Sect. 5.

## The Reaction–Diffusion Master Equation

Consider the following chemical reaction system:2This denotes that a molecule of type *A* can bind to a molecule of type *B* to create a molecule of type *C* and that this process occurs with rate $k_1$. Furthermore, a molecule of type *C* can spontaneously unbind into one molecule of type *A* and one molecule of type *B*, a process which occurs with rate $k_2$. As mentioned in Introduction, the simplest method of modelling systems like (2) is to describe the concentrations (number of molecules per unit volume) of *A*, *B* and *C* as differentiable functions of time, denoted [*A*], [*B*] and [*C*], respectively. These functions are implicitly defined as the solutions to a set of ordinary differential equations, the REs:

3 $$\begin{aligned} \frac{\hbox {d}[A]}{\hbox {d}t}&=-\,k_1[A][B]+k_2[C],\nonumber \\ \frac{\hbox {d}[B]}{\hbox {d}t}&=-\,k_1[A][B]+k_2[C],\nonumber \\ \frac{\hbox {d}[C]}{\hbox {d}t}&=k_1[A][B]-k_2[C]. \end{aligned}$$

The equation for $\frac{\hbox {d}[A]}{\hbox {d}t}$, for example, states that the rate of change of [*A*] is equal to minus the rate at which *A* molecules are removed from the system (the rate of the binding reaction, $k_1$, multiplied by the product of concentrations of the reactants, [*A*][*B*]) plus the rate at which *A* molecules are added to the system (the rate of the unbinding reaction, $k_2$, multiplied by the concentration of the reactant, [*C*]). The functional form of these rates follows from the principle of mass action kinetics (Erdi and Janos 1989), which states that the rate of a reaction is proportional to the product of concentrations of the reactants.

Equation (3), coupled with initial concentrations of each species, provides all the information required to ascertain the concentrations of *A*, *B* and *C* at any time in the future, either by using a numerical ODE solver (Butcher 2016), or by analytically solving the equations.

The REs are simple to derive for any system, simple to understand, and easy to solve with a computer, and therefore they remain by far the most common model of chemical kinetics in use today, in fields ranging from physical chemistry (Rickard 1997) to cell biology (Schnell and Mendoza 1997).

However, the RE model relies heavily on the assumption that concentrations are differentiable functions of time, which is clearly untrue since numbers of molecules must be integer-valued, and therefore discrete. This assumption becomes particularly egregious when the concentrations are small, so that a system may contain only a few tens or hundreds of molecules of each species. In addition to this, it must be noted that chemical kinetics is inherently probabilistic, for a variety of reasons. For one, chemical reactions are quantum mechanical events (Atkins et al. 2018), though the details of this are beyond the scope of this article. Another reason is that bimolecular reactions occur only when the two reactants diffuse close enough together to react, and diffusion (the cumulative effect of huge numbers of collisions with water molecules) is such a complex process that it is typically modelled as random (Gillespie and Seitaridou 2012).

Thinking along the lines of discreteness and randomness leads us to consider not concentrations [*A*], [*B*] and [*C*], but rather the joint probability mass function $P(n_A,n_B,n_C;t)$, the probability that the system contains exactly $n_A$ molecules of *A*, $n_B$ molecules of *B* and $n_C$ molecules of *C* at time *t*. Though this quantity may seem hopelessly complicated, it turns out that we can say quite a lot about it.

Consider a very short time period $\Delta t$, so short that at most one reaction can happen in it, then consider what we can say about $P(n_A,n_B,n_C;t+\Delta t)$ in terms of $P(n_A,n_B,n_C;t)$. We can end up with exactly $n_A,n_B,n_C$ molecules at time $t+\Delta t$ in three different ways: (I) if there were $n_A+1,n_B+1,n_C-1$ molecules at time *t* and a binding reaction happened in the interval $[t,t+\Delta t)$; (II) if there were $n_A-1,n_B-1,n_C+1$ molecules at time *t* and an unbinding reaction happened in $[t,t+\Delta t)$; and (III) if there were $n_A,n_B,n_C$ molecules at time *t* and no reactions happened in $[t,t+\Delta t)$. So we can write:

4 $$\begin{aligned} P(n_A,n_B,n_C;t+\Delta t)=P(\text {I})+P(\text {II})+P(\text {III}), \end{aligned}$$

where *P*(*i*) represents the probability that scenario (*i*) happened. By the definition of conditional probability (Loeve 1977), we can write the following for $P(\text {I})$:

5 $$\begin{aligned} P(I)&=P(n_A+1,n_B+1,\nonumber \\&\quad n_C-\,1 \text { molecules at time }t \text { and a binding reaction happened}) \end{aligned}$$

6 $$\begin{aligned}&=P(n_A+1,n_B+1,n_C-1;t)\nonumber \\&\quad \times \, P(\text {a binding reaction happened in time }\Delta t ~\vert ~ n_A+1,\nonumber \\&\quad n_B+\,1,n_C-1 \text { molecules at time }t), \end{aligned}$$

where the conditional symbol $\vert$ means “given that”.

At this point in the analysis we need to make an assumption, namely that the waiting times between chemical reactions are exponentially distributed. For unimolecular reactions (i.e. unbinding), Fermi’s golden rule implies that waiting times are very close to exponential (Dirac 1927; Fermi 1950). For bimolecular reactions (i.e. binding) the reality is more complicated: reacting molecules must diffuse close together, then collide with a sufficiently high energy, at the correct orientation (Atkins et al. 2018). Although the underlying processes involved here are ultimately deterministic (at least at the level we go to in this article), they are so complex that they appear to be random, and the overall waiting time will appear to follow some probability distribution. The exponential distribution is chosen because of the assumption of *memorylessness*, meaning that the future of the system depends only on the current state (i.e. molecule numbers) and not on the states which preceded it. Exponential waiting times are a direct consequence of this assumption. Though it is a straightforward assumption, it is not strictly true, for instance, imagine that a system is currently in the state $(n_A=10,~n_B=10,~n_C=10)$. According to the memorylessness assumption, the probability that a binding reaction happens next is independent of how the system got into its current state, whether from state $(n_A=11,~n_B=11,~n_C=9)$ and a binding reaction, or from state $(n_A=9,~n_B=9,~n_C=11)$ and an unbinding reaction. Yet if an unbinding reaction happened very recently, then we know that the products of that reaction will be close together, and so will be significantly more likely to bind than a typical pair of reactants. In other words, binding reactions are more likely in the immediate aftermath of an unbinding, violating the memorylessness assumption. Choosing the exponential distribution essentially amounts to ignoring this rather subtle effect, but in its favour the exponential distribution has very useful mathematical properties.

One of the nice mathematical properties of exponentially distributed events is that the probability of the event happening in a short interval $\Delta t$ is proportional to $\Delta t$. By the principle of mass action, this probability is also proportional to the number of molecules of the reactants. It follows that we can write:

7 $$\begin{aligned}&P(\text {a binding reaction happened in time }\Delta t~\vert ~ n_A+1,\nonumber \\&\qquad n_B+1,n_C-1 \text { molecules at time } t)\nonumber \\&\quad =\frac{k_1}{V}(n_A+1)(n_B+1)\Delta t, \end{aligned}$$

where *V* is the reaction volume.

Why do we write $\frac{k_1}{V}$ instead of just $k_1$? This is quite a subtle point of statistical physics (Van Kampen 1992), but it can be intuitively justified by a dimensional argument. Concentrations have units of *inverse volume*, so in order for Eq. (3) to be dimensionally consistent, $k_1$ must have units of *volume per time*, whereas $k_2$ simply has units of *inverse time*. The extra *V* is thus required to make Eq. (7) dimensionally consistent. It can generally be shown that reactions with *n* reactants will have their rates scaled by $V^{1-n}$ (Van Kampen 1992). This can be intuitively justified by observing that bimolecular reactions will tend to happen less frequently in larger volumes, since it is harder for particles to find each other.

Applying the same arguments to scenarios (II) and (III), we find that we can write Eq. (4) as:

8 $$\begin{aligned} P(n_A,n_B,n_C;t+\Delta t)&=\frac{k_1}{V}(n_A+1)(n_B+1)\Delta t P(n_A+1,n_B+1,n_C-1;t)\nonumber \\&\quad +\,k_2(n_C+1)\Delta t P(n_A-1,n_B-1,n_C+1;t)\nonumber \\&\quad +\,\left( 1-\frac{k_1}{V}n_An_B\Delta t-k_2n_C\Delta t\right) P(n_A,n_B,n_C;t). \end{aligned}$$

This equation simplifies very nicely to give:

9 $$\begin{aligned}&\frac{P(n_A,n_B,n_C;t+\Delta t)-P(n_A,n_B,n_C;t)}{\Delta t}\nonumber \\&=\frac{k_1}{V}\left[ (n_A+1)(n_B+1)P(n_A+1,n_B+\,1,\right. \nonumber \\&\quad \left. n_C-1;t) -n_An_BP(n_A,n_B,n_C;t)\right] \nonumber \\&\quad +\,k_2\left[ (n_C+1) P(n_A-1,n_B-\,1,\right. \nonumber \\&\quad \left. n_C+1;t)-n_C P(n_A,n_B,n_C;t)\right] . \end{aligned}$$

The left-hand side of Eq. (9), in the limit of small $\Delta t$, is the definition of a derivative, and the right-hand side has no $\Delta t$ dependence, so we get:

10 $$\begin{aligned} \frac{\hbox {d}}{\hbox {d}t}P(n_A,n_B,n_C;t)&=\frac{k_1}{V}\left[ (n_A+1)(n_B+1) P(n_A+1,n_B+\,1,\right. \nonumber \\&\quad \left. n_C-1;t)-n_An_BP(n_A,n_B,n_C;t)\right] \nonumber \\&\quad +\,k_2\left[ (n_C+1) P(n_A-1,n_B-\,1,n_C+1;t)\right. \nonumber \\&\quad \left. -n_C P(n_A,n_B,n_C;t)\right] , \end{aligned}$$

which is known as the chemical master equation (CME) (Van Kampen 1992). Analogously to the REs, the CME can be solved numerically (Munsky and Khammash 2006), given an initial probability mass function $P(n_A,n_B,n_C;0)$, or it can be solved analytically if it is sufficiently simple (Darvey et al. 1966; Gadgil et al. 2005; Jahnke and Huisinga 2007; Shahrezaei and Swain 2008; Grima et al. 2012; Smith and Shahrezaei 2015), which this example is (Darvey et al. 1966; Van Kampen 1976; Cianci et al. 2016). A less intensive approach is to approximate either the distribution (Thomas and Grima 2015; Smith et al. 2015; Andreychenko et al. 2017; Smith and Grima 2017b), or the moments (e.g. the mean or variance) of $P(n_A,n_B,n_C;t)$, and a variety of methods are popular such as the van Kampen approximation (Van Kampen 1992; Elf and Ehrenberg 2003), moment-closure approximations (Gillespie 2009b; Singh and Hespanha 2011; Grima 2012; Schnoerr et al. 2014a, 2015), or the chemical Langevin equation (Gillespie 2000; Schnoerr et al. 2014b). For a recent review see Ref. Schnoerr et al. (2017).

There is an alternative (and much more popular) approach to the CME, which has no analogue corresponding to the REs. This approach notes that $P(n_A,n_B,n_C;t)$ is not just any function, but is a probability distribution, and therefore, pseudo-random samples can be drawn from it exactly as one might sample from a Gaussian or a Poisson distribution (LEcuyer 2012). In fact, $P(n_A,n_B,n_C;t)$ is actually an infinite set of related probability distributions indexed by *t*, and so a sample will be an entire trajectory of molecule numbers over time. Such trajectories are much more intuitive than the CME itself, because each trajectory represents a particular realisation of what we might actually see if we observed a system in real time. Furthermore, it turns out it is typically computationally much easier to sample from the CME than to solve the CME, and if we take a large number of sample trajectories we can use them to estimate $P(n_A,n_B,n_C;t)$. The reason for this computational difference is that the cost of numerical solution of the CME scales with the number of possible system states (i.e. the number of permissible combinations of molecule numbers), while the cost of sampling scales with the frequency of reactions. Numerically solving the CME will typically only be worthwhile for systems with a small number of states and fast reaction rates.

Sampling trajectories of the CME is typically referred to as “simulating” the underlying system, for obvious reasons. The most common way to simulate is to use Gillespie’s stochastic simulation algorithm (SSA) (Gillespie 1977), because this algorithm gives statistically exact trajectories, by taking advantage of the fact that the waiting times between reactions are exponentially distributed. The SSA can be quite slow, because it explicitly simulates every reaction, so a huge number of approximate simulation algorithms have been developed. The most popular is a time-discretised algorithm called $\tau$-leaping, also by Gillespie (2001), but new algorithms are proposed every year that typically sacrifice a degree of accuracy for an increase in speed (Salis and Kaznessis 2005; Cao et al. 2005; Auger et al. 2006). For a recent review see Ref. Szekely and Burrage (2014).

**Fig. 2:**
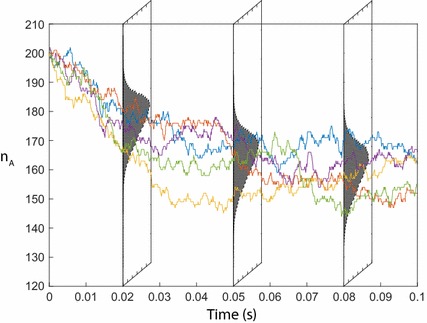
Stochastic simulations and analytical solutions of Eq. (10). The five multicoloured trajectories are independent simulations made using the SSA. Exact probability distributions solving Eq. (10) are shown as perpendicular grey histograms at $t=0.02$, 0.05 and 0.08. This makes clear that at each time point *t* the numerical value of each trajectory is a sample from the probability distribution solving the CME

The relationship between individual sample trajectories and the CME solution $P(n_A,n_B,n_C;t)$ is quite unintuitive, so we have shown an example in Fig. 2. The main graph shows five independent sample trajectories of the CME Eq. (10), showing the number of *A* molecules $n_A$ over a short time period. Perpendicular to the main graph are three histograms showing the marginal probability distribution $P(n_A;t)$ at three time points $t=0.02$, 0.05 and 0.08. This representation makes it clear that the values of the trajectories of $n_A$ at $t=0.02$ (for example) are independent samples of the distribution $P(n_A;0.02)$. There is a one-to-one correspondence between the distributions $P(n_A,n_B,n_C;t)$ and the sample trajectories: if we know the distributions then we can sample trajectories; if we have enough independent trajectories we can approximate the distributions to an arbitrarily high degree of accuracy.

One point that we have not satisfactorily addressed so far is the validity of the principle of mass action, the principle which underpins fundamental equations such as (7). The principle of mass action is based on combinatorial arguments: if a reaction has two reactants, *A* and *B* say, then the probability of a reaction must be proportional to the number of ways a reacting pair can be made up, i.e. $n_An_B$ different ways; similarly, if a reaction involves two molecules of *A* reacting with each other, then we would expect the reaction probability to scale as $\frac{n_A(n_A-1)}{2}$.

**Fig. 3:**
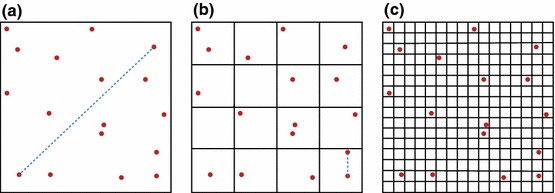
The rationale behind dividing a volume into subvolumes. **a** The principle of mass action claims that every reacting pair of molecules is equally likely to react in any time period, including pairs which might be very distant (blue dotted line). **b** One solution is to divide the volume into *M* subvolumes, and only allow molecules in the same subvolume to react, so reacting pairs are now close together (blue dotted line). **c** If *M* is too large, then there will very rarely be pairs in the same subvolume, and bimolecular reactions will tend not to happen at all

These arguments are not particularly satisfactory when considered from a microscopic point of view: if no pair of molecules is sufficiently close together to collide and react in a short time $\Delta t$, then a reaction will not happen, no matter how many molecules there are overall; or if only 2 (or 3 or 4 or ...) pairs are sufficiently close together, then the probability of reaction will be roughly proportional to 2 (or 3 or 4 or ...) rather than the total number of pairs. The principle of mass action surely amounts to assuming (nonsensically) that *all* the pairs are sufficiently close together to potentially react in a short time $\Delta t$. The absurdity of this assumption is demonstrated graphically in Fig. 3a. A counter-argument to this is that we have no way of knowing how many pairs are close together, so we stick to assuming that a fixed proportion of them are close together and absorb the proportionality constant into the reaction rate. But this again is not truly satisfactory because the true proportion of pairs that are close together is itself a random variable and not a constant.

It turns out that the principle of mass action is closely related to the *diffusion* of the reacting molecules. The diffusion coefficient is essentially a measure of the rate at which a randomly diffusing particle moves around the reaction volume. A particle with a very high diffusion coefficient could have diffused all over the volume in a short time $\Delta t$, whereas a particle with a small diffusion coefficient may only have covered a small region of space. (The average distance covered by a diffusing molecule before it reacts is known as the *Kuramoto length*, see Ref. Grima and Schnell 2008.) It follows that the principle of mass action could plausibly apply if particles have very high diffusion coefficients, so that any pair could potentially collide in a short time $\Delta t$. This idea can actually be proved from a microscopic point of view (and we will demonstrate this later), but *only if* we assume all particles have infinite (or practically infinite) diffusion coefficients. (Of course, the concept of an “infinite diffusion coefficient” is physically impossible, and we will address this point shortly.) Doubting the principle of mass action is then essentially equivalent to believing that the diffusion coefficients might not be high enough for the reaction volume to be well mixed, and this is a sensible belief as far as cell biology is concerned (Wojcieszyn et al. 1982). Clearly, an alternative approach is needed: in particular, we want a model which can tell us how many pairs of particles are “close together” at any given time.

The simplest way to go about this is to subdivide the entire reaction volume into small subvolumes, with the implication that we will consider molecules in the same subvolume to be “close together”, while molecules in different subvolumes are not. Suppose we have chosen to divide our volume into *M* subvolumes, which for simplicity we assume are equally sized; then, the probability of a reaction involving *A* and *B* in subvolume *i* will be proportional to $n_A^{(i)}n_B^{(i)}$, where $n_X^{(i)}$ denotes the number of molecules of species *X* in subvolume *i*. This idea is shown in Fig. 3b.

While superficially satisfying, this description actually raises more questions than it answers, at least at first. Principally, how should we choose *M*? Clearly, we should choose $M>1$; otherwise, the description is identical to the CME, and generally, if *M* is small we will tend to have the same issues with the principle of mass action that the CME had, which drove us to seek a new model. On the other hand, if we make *M* very large, the subvolumes will become very small (possibly smaller than the physical size of a molecule) and the probability of two reacting molecules being in the same subvolume at a given time becomes negligible. As shown in Fig. 3c, this results in bimolecular reactions simply not happening, which is clearly something we would like to avoid.

Choosing the correct partition of the volume into subvolumes requires us to be more specific about what we truly mean by “close together”, a phrase about which we have thus far been deliberately vague. To do this, we would need to propose a physical model for how chemical reactions occur, including molecular sizes and shapes, how they diffuse, whether they react immediately upon collision or whether they need a sufficiently large kinetic energy, and how electrostatic and hydrodynamic interactions impact on the reaction. These are questions that go far beyond the scope of our models, which would significantly detract from their simplicity. As a result, the standard response is to be vague about the value of *M*, suggesting that it should be neither too small nor too large. Though this is not a very satisfying answer, it highlights that there is no value of *M* which can be pre-specified for all situations: the correct *M* will depend on a number of factors and will likely be different for different situations, such as different diffusion coefficients.

Another significant issue with this model is how we decide how many molecules are in the *i*th subvolume. This is an issue because molecules, in reality, do not remain in the same location forever, but diffuse throughout the reaction volume—indeed, it was the issue of diffusion which led us to seek out a new model. To address this issue, drawing on the argument in Ref. Gillespie et al. (2014), we will consider a simplified one-dimensional volume and a single diffusing molecule currently located at a point *x*. It is well known that the probability density functions of diffusing point-particles obey a partial differential equation (PDE) called the diffusion equation:

11 $$\begin{aligned} \frac{\partial }{\partial t} p(x,t)=D\frac{\partial ^2}{\partial x^2}p(x,t), \end{aligned}$$

where *p*(*x*, *t*) is the probability density of finding the particle at location *x* at time *t*, and *D* is the particle’s diffusion coefficient. We can rewrite Eq. (11) in terms of small increments $\Delta t$ and $\Delta x$ and rearrange to get:

12 $$\begin{aligned} p(x,t+\Delta t)=p(x,t)+\frac{D\Delta t}{\Delta x^2}\left[ p(x+\Delta x,t)-2p(x,t)+p(x-\Delta x,t)\right] . \end{aligned}$$

Now, consider a partition of the total volume into *M* subvolumes of incremental width $\Delta x$, and let subvolume *k* be centred around the point *x*. If we denote by *q*(*k*, *t*) the probability that the molecule is in subvolume *k* at time *t*, then we will have that:

13 $$\begin{aligned} q(k,t)=\int _{x-\Delta x/2}^{x+\Delta x/2}p(y,t)\hbox {d}y\approx p(x,t)\Delta x, \end{aligned}$$

where the approximation will tend to hold when $\Delta x$ is small. It follows that we can write:

14 $$\begin{aligned} q(k,t+\Delta t)=q(k,t)+\frac{D\Delta t}{\Delta x^2}\left[ q(k+1,t)-2q(k,t)+q(k-1,t)\right] . \end{aligned}$$

In other words, in a short time $\Delta t$, the probability that the molecule moves from subvolume *k* to subvolume $k+1$ is equal to $\frac{D\Delta t}{\Delta x^2}$. Equation (14) has the form of a master equation, which implies that the waiting time for a molecule diffusing between subvolumes is approximately exponential, at least when the subvolumes are small, but it is a reasonable approximation even when the subvolumes are quite large.

This leads us to a model in which particles “hop” between neighbouring subvolumes at random times, and the waiting times between hoppings are exponentially distributed. This means that we can represent hopping events as just another type of reaction event. This seems a little counter-intuitive, but makes sense when studied in detail. Let us consider system (2) again under this model. We can write $X^{(i)}$ to denote the species *X* in subvolume *i*. Then we can write the new system in the following way:15where $k_D^{(X,i,j)}$ is the “hopping rate” at which a molecule of type *X* in subvolume *i* will hop into subvolume *j*, which will be equal to zero if *i* and *j* are not neighbouring subvolumes. Note that we do not use the specific rate obtained in Eq. (14), because that rate is derived assuming that the subvolumes are arranged in a particular regular manner, and we would like to retain generality for the time being. Clearly, system (15) is much more complicated than (2), but there is little actual difference in principle. Both systems comprise species, reactions and rates—it just happens that the “species” in system (15) are not just the types of molecules (*A*, *B*, *C*), but the types of molecules in a particular subvolume ($A^{(i)}$, $B^{(i)}$, $C^{(i)}$). This might be unintuitive, but it makes no difference mathematically. It follows, then, that any technique which can be applied to system (2) can also be applied to system (15).

For example, analogously to the REs (3), we can write equations for the concentrations $[A^{(i)}]$, $[B^{(i)}]$ and $[C^{(i)}]$ (intuitively, the local concentrations of *A*, *B* and *C* in subvolume *i*):

16 $$\begin{aligned} \frac{\hbox {d}[A^{(i)}]}{\hbox {d}t}&=-\,k_1[A^{(i)}][B^{(i)}]+k_2[C^{(i)}] +\sum _{j=1}^M\left[ -k_D^{(A,i,j)}[A^{(i)}]+k_D^{(A,j,i)} [A^{(j)}]\right] ,\nonumber \\ \frac{\hbox {d}[B^{(i)}]}{\hbox {d}t}&=-\,k_1[A^{(i)}][B^{(i)}]+k_2[C^{(i)}] +\sum _{j=1}^M\left[ -k_D^{(B,i,j)}[B^{(i)}]+k_D^{(B,j,i)} [B^{(j)}]\right] ,\nonumber \\ \frac{\hbox {d}[C^{(i)}]}{\hbox {d}t}&=k_1[A^{(i)}][B^{(i)}]-k_2[C^{(i)}] +\sum _{j=1}^M\left[ -\,k_D^{(C,i,j)}[C^{(i)}]+k_D^{(C,j,i)} [C^{(j)}]\right] , \end{aligned}$$

where, as before, *M* is the number of subvolumes. Equation (16) is the analogue of the REs with spatial resolution, but it would be helpful if we had a better idea of the form of the hopping rates $k_D^{(X,i,j)}$. Calculating the hopping rates from microscopic principles is an extremely challenging problem and, in fact, is still an open research question. In the simplest case, where each subvolume is an equally sized cube (or square, or line segment) of side length *h* arranged in a Cartesian grid, then we have already seen that there is a simple expression:

17 $$\begin{aligned} k_D^{(X,i,j)}=\left\{ \begin{array}{ll} \frac{D_X}{h^2}&{}\quad \text {if }\;i\ \text {neighbours }j,\\ 0&{}\quad \text {otherwise,} \end{array} \right. \end{aligned}$$

where $D_X$ is the diffusion coefficient associated with particles of type *X*.

If we imagine a one-dimensional array of subvolumes of equal size arranged in a line of length *L*, such that subvolume 1 neighbours subvolume 2, subvolume 2 neighbours subvolumes 1 and 3, subvolume 3 neighbours 2 and 4, etc., then Eq. (16) becomes:

18 $$\begin{aligned} \frac{\hbox {d}[A^{(i)}]}{\hbox {d}t}&=-\,k_1[A^{(i)}][B^{(i)}]+k_2[C^{(i)}] +\frac{D_A}{(L/M)^2}\left[ [A^{(i-1)}]-2[A^{(i)}]+[A^{(i+1)}]\right] ,\nonumber \\ \frac{\hbox {d}[B^{(i)}]}{\hbox {d}t}&=-\,k_1[A^{(i)}][B^{(i)}]+k_2[C^{(i)}] +\frac{D_B}{(L/M)^2}\left[ [B^{(i-1)}]-\,2[B^{(i)}]+[B^{(i+1)}]\right] ,\nonumber \\ \frac{\hbox {d}[C^{(i)}]}{\hbox {d}t}&=k_1[A^{(i)}][B^{(i)}]-k_2[C^{(i)}] +\frac{D_C}{(L/M)^2}\left[ [C^{(i-1)}]-2[C^{(i)}]+[C^{(i+1)}]\right] , \end{aligned}$$

for $i=2,\ldots ,M-1$ with small modifications for the end subvolumes 1 and *M* depending on the boundary conditions. We can now imagine taking the limit $M \rightarrow \infty$. There are a couple of issues with this. First, we already noted that choosing subvolumes too small could be a problem because the probability of two molecules being in the same subvolume would become negligibly small, thus making bimolecular reactions unusually rare events. We will bypass this issue by saying that the limit $M\rightarrow \infty$ is simply an approximation, which we expect to be accurate when concentrations are high. The second issue is that the notation $[A^{(i)}]$ becomes meaningless when *M* is infinite. This we will solve by replacing $[A^{(i)}]$ with [*A*] which we consider to be a function of location $x=\frac{iL}{M}$ as well as time. In the limit $M \rightarrow \infty$, *x* approaches a continuous quantity, so that differentiation with respect to *x* becomes valid. For example, $\frac{D_A}{(L/M)^2}\left[ [A^{(i-1)}]-2[A^{(i)}]+[A^{(i+1)}]\right]$ converges to $D_A\frac{\partial ^2 [A]}{\partial x^2}$ in the limit $M \rightarrow \infty$. As a result, we get the following PDEs:

19 $$\begin{aligned} \frac{\partial [A]}{\partial t}&=-\,k_1[A][B]+k_2[C]+D_A\frac{\partial ^2 [A]}{\partial x^2},\nonumber \\ \frac{\partial [B]}{\partial t}&=-\,k_1[A][B]+k_2[C]+D_B\frac{\partial ^2 [B]}{\partial x^2},\nonumber \\ \frac{\partial [C]}{\partial t}&=k_1[A][B]-k_2[C]+D_C\frac{\partial ^2 [C]}{\partial x^2}. \end{aligned}$$

These PDEs are the well-known reaction–diffusion equations (RDEs), ubiquitous in mathematical biology (Murray 2001), and popularised by Alan Turing in his seminal paper Ref. Turing (1952). Turing famously demonstrated that certain systems (Gierer and Meinhardt 1972) could be unstable when modelled with the RDEs, but stable when modelled with the REs: this kind of instability manifests itself as visual patterns (e.g. spots, stripes) in an RDE simulation. The Turing instability (as it is now known) is believed by some to be the cause of biological patterns such as zebrafish stripes (Nakamasu et al. 2009) or the regular spacing between mammalian digits (fingers and toes) (Sheth et al. 2012; Raspopovic et al. 2014), but there is still controversy around whether the Turing mechanism is really behind these phenomena (Watanabe and Kondo 2015). One of the current biggest challenges in synthetic biology is therefore to synthesise a Turing patterning network in living cells (Lengyel and Epstein 1992; Diambra et al. 2014; Borek et al. 2016; Scholes and Isalan 2017; Smith and Dalchau 2018a, b), which would provide convincing evidence of Turing’s theory.

As well as the REs, we can also apply the CME methodology to system (15). Instead of the probability mass function $P(n_A,n_B,n_C;t)$, we now consider a new probability mass function $P(\vec {n}_A,\vec {n}_B,\vec {n}_C;t)$, where $\vec {n}_X=(n_X^{(1)},\ldots ,n_X^{(M)})$ is a vector of molecule numbers, with one entry for each subvolume. Because of the complexity of the RDME, we have to introduce some new notations to be able to write it down compactly: we let $E_{(X,i)}$ be the shift operator which replaces any instance of $n_X^{(i)}$ with $n_X^{(i)}+1$. For example: $E_{(X,i)}n_X^{(i)}=n_X^{(i)}+1$, and, $E_{(X,i)}^{-1}n_X^{(i)}=n_X^{(i)}-1$, and for any function $f(\cdot )$, $E_{(X,i)}f(n_X^{(i)})=f(n_X^{(i)}+1)$. Note that the inverse operator $E_{(X,i)}^{-1}$ replaces $n_X^{(i)}$ with $n_X^{(i)}-1$.

Following the argument for the CME, we consider a time-step $\Delta t$, sufficiently short that at most one reaction (including hopping events) can occur. Then, we consider the ways we can end up with $\vec {n}_A,\vec {n}_B,\vec {n}_C$ molecules at time $t+\Delta t$. This can happen if: (I) a binding reaction happens in subvolume *i*, for some $i=1,\ldots ,M$, in the time interval $[t,t+\Delta t)$; (II) an unbinding reaction happens in subvolume *i*, for some $i=1,\ldots ,M$, in $[t,t+\Delta t)$; (III) a particle of type *X* hops from subvolume *i* to subvolume *j*, for some $i,j=1,\ldots ,M$ and $X\in \{A,B,C\}$, in $[t,t+\Delta t)$; or (IV) no reactions happen in the time interval $[t,t+\Delta t)$. Clearly, this is much more complicated than for the CME, but exactly the same principles apply, and after some simplifications we obtain the following equation:

20 $$\begin{aligned} \frac{\hbox {d}}{\hbox {d}t}P(\vec {n}_A,\vec {n}_B,\vec {n}_C;t)&=\sum _{i=1}^M \frac{k_1M}{V}\left[ E_{(A,i)}E_{(B,i)}E_{(C,i)}^{-1}-1\right] n_A^{(i)}n_B^{(i)}P(\vec {n}_A,\vec {n}_B,\vec {n}_C;t)\nonumber \\&\quad +\,\sum _{i=1}^M k_2\left[ E_{(A,i)}^{-1}E_{(B,i)}^{-1}E_{(C,i)} -1\right] n_C^{(i)}P(\vec {n}_A,\vec {n}_B,\vec {n}_C;t)\nonumber \\&\quad +\,\sum _{i=1}^M\sum _{j=1}^Mk_D^{(A,i,j)}\left[ E_{(A,i)} E_{(A,j)}^{-1}-1\right] n_A^{(i)}P(\vec {n}_A,\vec {n}_B, \vec {n}_C;t)\nonumber \\&\quad +\,\sum _{i=1}^M\sum _{j=1}^Mk_D^{(B,i,j)}\left[ E_{(B,i)} E_{(B,j)}^{-1}-1\right] n_B^{(i)}P(\vec {n}_A,\vec {n}_B, \vec {n}_C;t)\nonumber \\&\quad +\,\sum _{i=1}^M\sum _{j=1}^Mk_D^{(C,i,j)}\left[ E_{(C,i)} E_{(C,j)}^{-1}-1\right] n_C^{(i)}P(\vec {n}_A,\vec {n}_B, \vec {n}_C;t), \end{aligned}$$

noting the correct volume scaling $\frac{k_1M}{V}$ for the bimolecular reaction. This equation is known as the reaction–diffusion master equation (RDME). The first two lines correspond to the two reactions in each subvolume, while the final three lines correspond to the hopping of particles of type *A*, *B* and *C*, respectively, between neighbouring subvolumes.

Now that we have the RDME (which is really just a special type of CME); we can do to it anything that we could do to the CME. For instance, it is possible to solve RDMEs analytically, if they are composed exclusively of certain types of linear reactions (Jahnke and Huisinga 2007; Gadgil et al. 2005) or purely reversible reactions (Cianci et al. 2016). The example (15) is actually solvable, as long as the hopping events are reversible (i.e. as long as $k_D^{(X,i,j)}=k_D^{(X,j,i)}$, for all $X=A,B,C$ and $i,j=1,\ldots ,M$). Other analytical approximation techniques for the CME which are beyond the scope of this article [e.g. the van Kampen approximation (1992) and the chemical Langevin equation (Gillespie 2000)] can also be applied to the RDME, and have been, with interesting results (Smith et al. 2016; Ghosh et al. 2015).

Any stochastic simulation algorithm designed for the CME can also naturally be applied to the RDME (Stundzia and Lumsden 1996; Elf and Ehrenberg 2004; Bernstein 2005; Fange et al. 2012). Several new issues arise when simulating the RDME, however. For example, stochastic simulation algorithms tend to have computation time proportional to the frequency of reaction events (Gillespie 1977). If the hopping rates are quite large, then an RDME simulation may take substantially longer than an equivalent CME simulation—it is not uncommon for upwards of 99% of the simulation time to be spent simulating hopping events rather than chemical reactions. This issue in particular has driven a huge amount of research into fast and accurate simulation of the RDME (Elf et al. 2003; Drawert et al. 2010; Roberts et al. 2013; Fu et al. 2014).

**Fig. 4:**
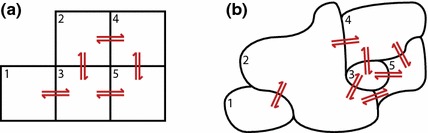
**a** If subvolumes are arranged in a grid, all hopping rates are straightforwardly given by Eq. (17). **b** If subvolumes are of irregular shapes, and boundaries between subvolumes are of different sizes, it is not clear how to best choose hopping rates

It is worth going into a little more detail now about the hopping rates $k_D^{(X,i,j)}$. In the simplest case where each subvolume is of the same size, Eq. (17) can be used. This case is demonstrated in Fig. 4a: neighbouring subvolumes (such as 1 and 3) all have the same hopping rates between them, whereas non-neighbouring subvolumes (such as 1 and 2) have zero hopping rates. More generally, subvolumes can be of any shape and size, and the size of the boundaries where two neighbouring subvolumes join can vary greatly as well. Such a case is shown in Fig. 4b: neighbouring subvolumes (such as 1 and 2) tend to be of different sizes, so the hopping rate in one direction will generally be different from the other; moreover, boundaries between neighbours are not necessarily of the same length (such as the boundaries between 2 and 4, and 2 and 5), and shorter boundaries will tend to be crossed by fewer molecules, and so should have correspondingly lower hopping rates. In these cases, the hopping rate should (in principle) be calculated using a first passage time approach with the diffusion equation (Redner 2001), but this is often computationally unfeasible and in practice there is no easy answer. Some authors have proposed techniques for choosing these rates in a manner which agrees optimally with Brownian diffusion (Isaacson and Peskin 2006; Engblom et al. 2009; Bayati et al. 2011; Drawert et al. 2012; Lotstedt and Meinecke 2015; Meinecke et al. 2016; Meinecke and Ltstedt 2016), but these are typically simulation-based and not analytically straightforward.

In this section, we typically assume that molecules do not occupy any volume themselves (i.e. they are point-particles), but relaxing this assumption leads to even more complex hopping rates. These rates typically depend on the concentrations of the various species in the destination subvolume *j*, so that a subvolume with a high concentration of molecules is less likely to be able to accommodate a new molecule hopping into it (Elderfield 1985; Baker et al. 2010; Fanelli and McKane 2010; Cianci et al. 2016). Writing the hopping rate as a linear function of the concentrations is a popular choice, but when concentrations are very high certain nonlinear functions have been shown to be more accurate (Meinecke 2017; Cianci et al. 2017; Smith et al. 2017).

When diffusion coefficients are large, simulations of the RDME can become very slow (because molecules will hop around many times between reactions) (Drawert et al. 2010), and significant modifications to the hopping rates have been introduced to address this issue. In particular, it is common to allow particles to hop between non-neighbouring subvolumes *i* and *j* (Taylor et al. 2014). The implication is that the particle did in fact follow a neighbour-to-neighbour path from *i* to *j*, but the intermediate steps are eliminated for speed: a very careful choice of the hopping rates is required in these cases to correctly model diffusion.

We have seen how the RDME was obtained as an extension of the CME, to address the problem of mass action kinetics. In this sense, the RDME is a *top-down* model, since it is not a simplification of a more detailed microscopic model like Brownian dynamics. The risk with top-down models is that the new components (the hopping rates, in the case of the RDME) could be added in a flawed manner, making the model inconsistent with the other well-established models. In short, the RDME is just the CME with some additional terms to explicitly model diffusion, but how do we know that these additional terms are correct?

This question has plagued users of the RDME for decades. A whole subgenre of the reaction–diffusion field is dedicated to comparing the RDME with microscopic models, including various types of Brownian dynamics and molecular dynamics, which are taken to be the “ground truth”. Almost invariably, because of the complexities of the models involved, it is stochastic simulations of models which are compared, rather than the actual equations which make up the models (Baras and Mansour 1996; Dobrzynski et al. 2007; Fange et al. 2010). As a result, the outcome of these comparisons is typically to suggest optimal values of the various RDME model parameters (reaction rates, hopping rates, number and size of subvolumes) to get best agreement with the “ground truth” model, for a particular reaction–diffusion system. Such comparisons are therefore very limited in their applicability: there is no reason to believe that the optimal hopping rates (for example) for one system will be the same for another system, even if they are quite similar.

An alternative (though much trickier) approach is to directly compare the RDME with the “ground truth” model by writing out equations for both and discerning to what extent they agree (Collins and Kimball 1949; Fange et al. 2010; Hellander et al. 2015). This kind of comparison will provide general rules about when models agree and will not generally be limited by system specifics. The challenge is that “ground truth” models tend to be very hard to write down, since they are very complex and are usually conceived as models for simulation rather than mathematical analysis. A pioneer in this kind of comparison is Isaacson, who has published several articles analytically comparing the RDME with a variety of “ground truth” models (Isaacson 2008, 2009, 2013). Two principal results emerge from Isaacson’s work. First, the RDME can be thought of as an approximation to a particular BD model known as the Smoluchowski model (1917) (more on this later), but there is no limit in which one converges to the other (Isaacson 2009). It can be rigorously proved that as *M* increases, the approximation first gets better, and then gets worse (while never actually showing perfect agreement), and it is hard to know in general which *M* is the optimal one. In a similar vein, Hellander et. al. have shown that there exists a hard upper limit on *M*, above which the RDME cannot agree with the Smoluchowski model because the subvolumes become unphysically smaller than the size of a molecule (Hellander et al. 2012). Isaacson’s second principal result is that the RDME can be modified to create a new model (known as the “convergent RDME”, or CRDME) in which molecules can react with molecules in different subvolumes, according to certain strict rules (Isaacson 2013). The CRDME was shown to converge precisely to another BD model known as the $\lambda -\rho$ model (Erban and Chapman 2009) (more on this later). Overall, it seems that the RDME never quite sits comfortably with BD, and potentially, this could call its accuracy into question.

But we take a slightly different view of the RDME to those who wish to compare it to a “ground truth” model. Considering the RDME as an approximation to a microscopic model necessarily involves questions about molecular shapes and sizes and about the physical chemistry of reactions, questions which take us far from the neat simplicity of the CME. The RDME was conceived as a version of the CME with spatial resolution, to address the question of how diffusion affects chemical systems. Users of the RDME are not necessarily interested in (or even aware of) the size and shape of the molecules they are modelling, and to require knowledge of these quantities to correctly parameterise the RDME is a potential overcomplication. In our view, the RDME is a neat and straightforward model for *qualitatively* understanding the effect of diffusion on stochastic reaction systems, an important academic question for which no other model is well suited, but it is probably not appropriate to answer questions where numerical accuracy are paramount, and in these cases a microscopic model may be more suitable.

We do not worry particularly about the RDME’s agreement with more complex models, to the extent that we do not discredit it as a model simply because it disagrees with more microscopic models, but in Sect. 4 we do try to analyse *why* the RDME differs from BD. However, it is very important that the RDME should agree with the simpler models on the complexity scale in the conditions under which those models are accurate. In particular, we would like the RDME to agree with the CME when diffusion is very fast (i.e. when spatial resolution becomes unimportant); we would like the RDME to agree with the RDEs when concentrations are very high (i.e. when stochasticity becomes unimportant); and we would like it to agree with the REs when both of the above conditions hold (high concentrations and fast diffusion).

### The Limit of Fast Diffusion

We have previously mentioned the concept of “infinite diffusion”, which is somewhat counter-intuitive and is worth discussing. Diffusion coefficients in reality cannot be infinite. In the case of spherical particles, they are given by the Stokes–Einstein relation $D=\frac{k_BT}{6\pi \eta r}$, where $k_B$ is the Boltzmann constant, *T* is the temperature, $\eta$ is the viscosity of the solvent, and *r* is the particle radius (Gillespie and Seitaridou 2012). All of these quantities are finite and nonzero, so infinite diffusion is necessarily unphysical. When we say “infinite diffusion” we are not thinking of limiting case of small particle radii, low viscosity or high temperatures. Instead, we mean that the rate at which a particle moves between regions of space (the hopping rate, in the RDME) is much higher than the rate at which it is involved in chemical reactions (these conditions are typically referred to as “reaction-limited” (Grima and Schnell 2006), but thinking instead in terms of “infinite diffusion” will prove to be useful shortly). In other words, a typical particle will diffuse throughout the reaction volume several times before actually reacting. Under these conditions, the principle of mass action kinetics can reasonably be said to hold, since any pair of particles will have time to move close together before a reaction takes place, and the concept of particles being too far apart to react no longer makes sense. For this reason, we expect the CME to be an accurate model under the conditions of “infinite diffusion”, and so if the RDME is also accurate, it should agree with the CME in this case.

**Fig. 5:**
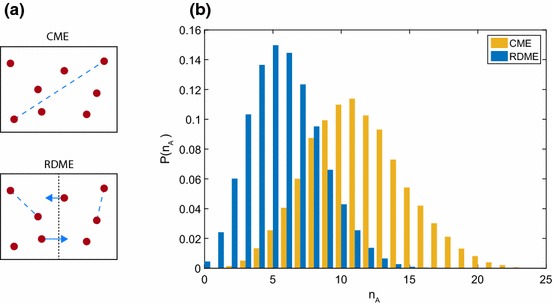
Comparing the fast diffusion limit of the RDME with the CME. **a** In the CME, any molecule (red) can react with any other (dashed blue line); in the RDME molecules can only react with other molecules in the same subvolume (dashed blue lines), but they can “hop” between neighbouring subvolumes (blue arrows). **b** The equilibrium distribution of the CME (yellow) and RDME with $M=2$ and fast diffusion ( $k_D=40$, blue) for system (34) obtained from stochastic simulations, with $k_1=0.1$, $k_2=1$, $K=100$ and $V=1$. If the RDME is correct these two distributions should essentially agree, but they are clearly very different. Note that the RDME distribution remains the same for larger $k_D$

To answer the question of agreement between the CME and RDME, we will study a straightforward but general example. For simplicity, we will consider a system consisting of only one chemical species, *A*, and two equally sized subvolumes inside a total volume of size *V*. The set-up is shown in Fig. 5a. The arguments that follow in fact apply to systems with any number of species and any number of subvolumes of any sizes, and a complete proof can be found in Ref. Smith and Grima (2016).

The system will consist of *R* reactions which take the form $s_j A\rightarrow r_j A$ for $j=1,\ldots ,R$. The rate of reaction *j* will be given by an arbitrary function $f_j(n_A,V)$. The CME of our system takes the form:

21 $$\begin{aligned} \frac{\hbox {d}}{\hbox {d}t}P(n_A;t)=\sum _{j=1}^R\left[ f_j(n_A+s_j-r_j,V) P(n_A+s_j-r_j;t)-f_j(n_A,V)P(n_A;t)\right] . \end{aligned}$$

In contrast, the RDME takes the form:

22 $$\begin{aligned} \frac{\hbox {d}}{\hbox {d}t}P\left( n_A^{(1)},n_A^{(2)};t\right)&=\sum _{j=1}^R\left[ f_j \left( n_A^{(1)}+s_j-r_j,\frac{V}{2}\right) P\left( n_A^{(1)} +s_j-r_j,n_A^{(2)};t\right) \right. \nonumber \\&\quad \left. -\,f_j\left( n_A^{(1)},\frac{V}{2}\right) P\left( n_A^{(1)},n_A^{(2)};t\right) \right] \nonumber \\&\quad +\,\sum _{j=1}^R\left[ f_j\left( n_A^{(2)}+s_j-r_j,\frac{V}{2}\right) P\left( n_A^{(1)},n_A^{(2)}+s_j-r_j;t\right) \right. \nonumber \\&\quad \left. -\,f_j\left( n_A^{(2)},\frac{V}{2}\right) P\left( n_A^{(1)},n_A^{(2)};t\right) \right] \nonumber \\&\quad +\,k_D^{(A,1,2)}\left[ \left( n_A^{(1)}+1\right) P\left( n_A^{(1)}+1,n_A^{(2)}-1;t\right) \right. \nonumber \\&\quad \left. -\,n_A^{(1)} P\left( n_A^{(1)},n_A^{(2)};t\right) \right] \nonumber \\&\quad +\,k_D^{(A,2,1)}\left[ \left( n_A^{(2)}+1\right) P\left( n_A^{(1)}-1,n_A^{(2)}+1;t\right) \right. \nonumber \\&\quad \left. -\,n_A^{(2)} P\left( n_A^{(1)},n_A^{(2)};t\right) \right] . \end{aligned}$$

We note that the second argument of the $f_j$ in the RDME is $\frac{V}{2}$ because each subvolume has volume $\frac{V}{2}$. Because each of the subvolumes is equal in size, and since they share a boundary, we can safely assume $k_D^{(A,1,2)}=k_D^{(A,2,1)}$. If this were not the case, it would imply a net flow of particles from one volume to the other, which may be appropriate for modelling some problems, but not the typical problem of diffusion which has no directional bias.

The limit of infinite diffusion implies that lines 3 and 4 of Eq. (22) will tend to overwhelmingly dominate, unless $P(n_A^{(1)},n_A^{(2)};t)$ has a form which makes lines 3 and 4 equal to zero. It follows that the system will converge to this form (the equilibrium distribution of lines 3 and 4) infinitely quickly, i.e. the following equation holds at all times:

23 $$\begin{aligned} 0&=k_D^{(A,1,2)}\left[ \left( n_A^{(1)}+1\right) P\left( n_A^{(1)}+1,n_A^{(2)}-1;t\right) -n_A^{(1)}P\left( n_A^{(1)},n_A^{(2)};t\right) \right] \nonumber \\&\quad +\,k_D^{(A,2,1)}\left[ \left( n_A^{(2)}+1)P(n_A^{(1)}-1,n_A^{(2)}+1;t\right) -n_A^{(2)}P\left( n_A^{(1)},n_A^{(2)};t\right) \right] . \end{aligned}$$

Since $k_D^{(A,1,2)}=k_D^{(A,2,1)}$, this simplifies further to:

24 $$\begin{aligned} 0&=\left( n_A^{(1)}+1\right) P\left( n_A^{(1)}+1,n_A^{(2)}-1;t\right) -n_A^{(1)} P\left( n_A^{(1)},n_A^{(2)};t\right) +\left( n_A^{(2)}+1\right) \nonumber \\&\quad \quad P\left( n_A^{(1)}-1, n_A^{(2)}+1;t\right) -n_A^{(2)}P\left( n_A^{(1)},n_A^{(2)};t\right) . \end{aligned}$$

This equation looks complicated, but in fact it has a straightforward non-trivial solution:

25 $$\begin{aligned} P\left( n_A^{(1)},n_A^{(2)};t\right) =\left\{ \begin{array}{ll} \frac{n_A!2^{-n_A}}{n_A^{(1)}!n_A^{(2)}!}&{}\quad \text {if }\;n_A^{(1)}+n_A^{(2)}=n_A,\\ 0&{}\quad \text {otherwise,} \end{array}\right. \end{aligned}$$

where $n_A$ is, as before, the total number of molecules of *A* in the entire volume. The solution (25) is a Binomial $\left( n_A,\frac{1}{2}\right)$ distribution, which is intuitively not surprising. If diffusion is infinitely fast, at any given time, each molecule has a $50\%$ chance of being in subvolume 1 and a 50% chance of being in subvolume 2. If there are $n_A$ molecules overall, then the solution must be the one given in Eq. (25).

We now take a moment to think about the meaning of the functions $f_j$. Since waiting times between reactions are exponentially distributed, $f_j(n_A,V)\Delta t$ is the probability that reaction *j* happens in a short time period of length $\Delta t$, given that there are $n_A$ molecules in the volume *V*. $f_j$ is precisely this probability in the CME, but the analogous expression for this probability in the RDME is not immediately obvious, because the RDME concerns the local molecule numbers $n_A^{(1)}$ and $n_A^{(2)}$ and the total molecule number $n_A$ is never explicitly modelled. Let us nonetheless denote it by $\bar{f}_j(n_A,V)$. It follows that the total molecule number in the RDME with infinitely fast diffusion satisfies the following equation:

26 $$\begin{aligned} \frac{\hbox {d}}{\hbox {d}t}P(n_A;t)=\sum _{j=1}^R\left[ \bar{f}_j(n_A+s_j-r_j,V) P(n_A+s_j-r_j;t)-\bar{f}_j(n_A,V)P(n_A;t)\right] , \end{aligned}$$

which is exactly the CME (21), but with $f_j$ replaced by $\bar{f}_j$. There is a subtle point here: we are saying that we can rewrite the RDME in terms of the total molecule numbers $n_A$, rather than the local molecule numbers $n_A^{(i)}$. We can do this because diffusion is infinitely fast, and so $n_A^{(i)}$ becomes a random variable parameterised by $n_A$, and the values of $n_A^{(i)}$ at any two distinct time points are uncorrelated. If diffusion were not infinitely fast, we would not be able to rewrite the RDME in the form of Eq. (26) without losing some information.

It remains to evaluate $\bar{f}_j$: if it is equal to $f_j$ then we have proved that the RDME converges to the CME in the limit of fast diffusion. $\bar{f}_j(n_A,V)\Delta t$ is the probability that reaction *j* happens in a time period $\Delta t$ and is equal to the probability that it happens in subvolume 1 or in subvolume 2. Since these events are independent, we get:

27 $$\begin{aligned} \bar{f}_j(n_A,V)\Delta t&=\sum _{n_A^{(1)}=0}^{n_A} \sum _{n_A^{(2)}=0}^{n_A}P\left( n_A^{(1)},n_A^{(2)};t\right) \left[ f_j\left( n_A^{(1)},\frac{V}{2}\right) \Delta t+f_j\left( n_A^{(2)},\frac{V}{2}\right) \Delta t\right] \nonumber \\&=2\sum _{n_A^{(1)}=0}^{n_A}\sum _{n_A^{(2)}=0}^{n_A} P\left( n_A^{(1)},n_A^{(2)};t\right) f_j\left( n_A^{(1)}, \frac{V}{2}\right) \Delta t\nonumber \\&=2\mathbb {E}\left[ f_j\left( n_A^{(1)},\frac{V}{2}\right) \Big \vert n_A\right] \Delta t, \end{aligned}$$

where the conditional expectation, $\mathbb {E}\left[ \star \vert n_A \right]$, denotes the expected value of $\star$ given that there are $n_A$ molecules overall. In short, $\bar{f}_j$ is proportional to the expected value of $f_j$ under the binomial distribution (25). We can try some example $f_j$’s to see what happens. For instance, if we choose $f_j(n_A,V)=k$ for some constant *k*, then we get:

28 $$\begin{aligned} \bar{f}_j=2\mathbb {E}\left[ k\vert n_A\right] =2k\ne k=f_j, \end{aligned}$$

so $f_j$ cannot simply be a constant. On the other hand, if we choose the correct volume scaling for a zero-order reaction propensity, $f_j(n_A,V)=kV$, then we get:

29 $$\begin{aligned} \bar{f}_j=2\mathbb {E}\left[ k\frac{V}{2}\vert n_A\right] =2k\frac{V}{2}=kV=f_j, \end{aligned}$$

so the RDME and CME are consistent. This shows the importance of the correct volume scaling for reaction rates. Some other standard rates are the monomolecular rate $f_j(n_A,V)=kn_A$, corresponding to a reaction of the form $A \xrightarrow {k}$, which gives:

30 $$\begin{aligned} \bar{f}_j=2\mathbb {E}\left[ kn_A^{(1)}\vert n_A\right] =2k\frac{n_A}{2}=kn_A=f_j, \end{aligned}$$

and the bimolecular rate $f_j(n_A,V)=\frac{k}{V}n_A(n_A-1)$, corresponding to a reaction of the form $A+A \xrightarrow {k}$, which gives:

31 $$\begin{aligned} \bar{f}_j=2\mathbb {E}\left[ \frac{2k}{V}n_A^{(1)}(n_A^{(1)}-1)\vert n_A\right] =\frac{4k}{V}\left( \frac{n_A}{4}+\frac{n_A^2}{4} -\frac{n_A}{2}\right) =\frac{k}{V}n_A(n_A-1)=f_j. \end{aligned}$$

These standard rates provide consistency between the RDME and CME, although the original physical reasoning behind these volume scalings have nothing to do with the RDME (Van Kampen 1992). But even rates which scale correctly with volume can have problems if they are unphysical in other ways. For instance, if we chose $f_j(n_A,V)=\frac{k}{V}n_A^2$, which has the correct volume scaling for a bimolecular reaction, we would get:

32 $$\begin{aligned} \bar{f}_j=2\mathbb {E}\left[ \frac{2k}{V}\left( n_A^{(1)}\right) ^2\vert n_A\right] =\frac{4k}{V}\left( \frac{n_A}{4}+\frac{n_A^2}{4}\right) =\frac{k}{V}n_A(n_A+1)\ne \frac{k}{V}n_A^2=f_j. \end{aligned}$$

Our RDME consistency criterion appears to very carefully select for rate functions with a sound physical basis, so it is clearly necessary to be very careful with rates before using the RDME.

While the “bad” rate functions given above are clearly just mistakes which can be rectified, there are some other genuine rate functions in common usage known as “non-elementary rates” which are not so fortunate. These include Hill function rates and Michaelis–Menten rates (Lawson et al. 2015). The Michaelis–Menten rate function has the form $f_j(n_A,V)=\frac{kVn_A}{KV+n_A}$, which gives:

33 $$\begin{aligned} \bar{f}_j= & {} 2\mathbb {E}\left[ \frac{kVn_A^{(1)}}{KV+2n_A^{(1)}}\vert n_A\right] =\frac{kVn_A ~_2F_1\left( \frac{KV}{2}+1,1-n_A; \frac{KV}{2}+2;-1\right) }{2^{n_A}\left( \frac{KV}{2}+1\right) }\nonumber \\\ne & {} \frac{kVn_A}{KV+n_A}=f_j, \end{aligned}$$

where $~_2F_1(a,b;c;d)$ is a hypergeometric function (Abramowitz and Stegun 1964). The RDME rate in this case is so different from the CME rate that it could give very wrong results if used. To check this, we simulated the following system:

34 $$\begin{aligned} \emptyset \xrightarrow {k_1} A,~ A \xrightarrow {MM}\emptyset , \end{aligned}$$

where $\emptyset$ denotes that we are not interested in the species involved, and *MM* denotes that the reaction occurs with the Michaelis–Menten rate $\frac{k_2Vn_A}{KV+n_A}$. System (34) was simulated using both the RDME with $M=2$ and fast diffusion, and the CME. The results are shown in Fig. 5. For the chosen parameters (see caption) the distributions are remarkably different: the RDME distribution has mean of around 7 compared with 11 for the CME. It is clear that using the RDME with non-elementary rates is a fundamentally bad idea. For more details of this effect, see Ref. Smith and Grima (2016).

We set out to demonstrate that the RDME is consistent with the CME when diffusion is infinitely fast, and we proved that this is the case but only under certain conditions. It appears that there is a strict subset of rate functions which are compatible with the RDME, and if a rate function is used which is not from this subset then the RDME is no longer a consistent model. This does not cast doubt on the RDME necessarily, but it does imply that extra caution should be taken when choosing rates for the RDME.

### The Limit of High Concentrations

As we have mentioned, the RDME is just a type of CME, and so any argument that holds for the CME will also hold for the RDME. The most fundamental result in the theory of stochastic chemical kinetics is that the CME converges to the REs when concentrations are high, thus implying that stochasticity stops being important for high concentrations. We will not go into the proof of this here, but it is discussed in detail in Refs. Kurtz (1971, 1972), Van Kampen (1992), Gardiner (1986), Ball et al. (2006) and Hepp et al. (2015). Naturally, this means that the RDME also converges to the spatially discretised analogue of the REs, Eq. (16), when concentrations are high. We will briefly offer a justification of this here, although it is not a rigorous proof by any means.

We will think about the number of *A* molecules in subvolume *i* at a time $t+\Delta t$, $n_A^{(i)}(t+\Delta t)$, conditional on the molecule numbers at time *t*. $n_A^{(i)}$ is typically a random variable, but when concentrations are high it will converge to its mean by the law of large numbers, so we can essentially treat it as a fixed value for now. Two facts help us to evaluate $n_A^{(i)}(t+\Delta t)$. Firstly, because there are huge numbers of molecules of all types in all subvolumes, a huge number of reaction events of each type will happen in the time period $[t,t+\Delta t)$. This implies that we do not need to think about the probability that a reaction happens, but the number of times it happens, and by the law of large numbers this number will converge (in the limit of high concentrations) to its expected value. Secondly, even though a huge number of reactions will happen in $[t,t+\Delta t)$, $\Delta t$ is still small, so the total molecule numbers will not change significantly over that period. This means that we can use $n_A^{(i)}(t)$ to refer to the value of $n_A^{(i)}$ at any point in the interval $[t,t+\Delta t)$. Following this argument, for system (2) in a volume *V* with length *L* divided linearly into *M* subvolumes, thinking about the rates of the processes which can increase or decrease $n_A^{(i)}$, we find that we can write:

35 $$\begin{aligned} n_A^{(i)}(t+\Delta t)= & {} n_A^{(i)}(t)-\frac{k_1M}{V}n_A^{(i)} (t)n_B^{(i)}(t)\Delta t+k_2n_C^{(i)}(t)\Delta t\nonumber \\&+\,\frac{D_AM^2}{L^2}\left[ n_A^{(i-1)}(t)-2n_A^{(i)}(t) +n_A^{(i+1)}(t)\right] \Delta t. \end{aligned}$$

Note that we have used the volume scaling $\frac{k_1M}{V}$ for the bimolecular reaction. Using the same arguments as in the derivation of the CME, Eq. (4), we can rearrange the $\Delta t$’s in Eq. (35) to produce a differential equation:

36 $$\begin{aligned} \frac{\hbox {d}}{\hbox {d}t}n_A^{(i)}=-\frac{k_1M}{V}n_A^{(i)}n_B^{(i)}+k_2n_C^{(i)} +\frac{D_AM^2}{L^2}\left[ n_A^{(i-1)}-2n_A^{(i)}+n_A^{(i+1)}\right] . \end{aligned}$$

We can divide through by the volume of the subvolumes $\frac{V}{M}$ to get the concentrations:

37 $$\begin{aligned} \frac{\hbox {d}}{\hbox {d}t}[A^{(i)}]=-\,k_1[A^{(i)}][B^{(i)}]+k_2[C^{(i)}] +\frac{D_AM^2}{L^2}\left[ [A^{(i-1)}]-2[A^{(i)}]+[A^{(i+1)}]\right] . \end{aligned}$$

Note that the volume scaling of the bimolecular rate disappears when we convert to concentrations. Equation (37) is exactly Eq. (18), which is the spatial analogue of the REs. We have already discussed that, in the limit of high concentrations, we can justifiably take the limit $M\rightarrow \infty$ to produce the RDEs Eq. (19), so the RDEs turn out to be the limiting form of the RDME when concentrations are high.

### The Combined Limits of Fast Diffusion and High Concentrations

We have established that the RDME reduces exactly to the CME in the limit of fast diffusion (if the rates are physically valid) or the RDEs in the limit of high concentrations; in this section, we will briefly consider what happens when *both* of these limits are applied simultaneously.

We have already noted that the high concentration limit of the CME is the REs, and we have a rough sketch of why this is the case. We will not go into the subject again here, and the proof can be found in Refs. Van Kampen (1992) and Gardiner (1986)

But what happens to the RDEs when diffusion is fast? The RDEs have the form:

38 $$\begin{aligned} \frac{\partial }{\partial t}[A]=R([A])+D_A\nabla ^2[A], \end{aligned}$$

where $R(\cdot )$ denotes reaction terms. When diffusion is fast, the second term of (38) dominates unless $\nabla ^2[A]=0$, which happens if [*A*] is a spatially uniform concentration. When diffusion is infinitely fast, the concentration [*A*] will converge to a spatially uniform concentration infinitely quickly. In that case, [*A*] will have no spatial dependence and will simply become a function of *t*. The RDEs then obey:

39 $$\begin{aligned} \frac{\partial }{\partial t}[A]=R([A]), \end{aligned}$$

which are simply the REs. It does not matter, therefore, in which order the limits of fast diffusion and high concentrations are applied: either way the RDME becomes the REs.

**Fig. 6:**
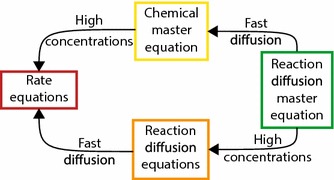
How the RDME fits into the scale of model complexity shown in Fig. 1. There is no direct relationship between the RDME and any of the more complex models

Overall, we have found that the RDME can be thought of as a “parent model” of the CME, the RDEs and the REs, with the relationship between them shown in Fig. 6. Specifically, if we use both the RDME and the CME (for example) to model the same system, and the system is such that we expect the CME to be correct (i.e. diffusion is fast), then the two models will give identical predictions. This is great for the RDME and demonstrates its place in the complexity scale (Fig. 1), but the RDME is nonetheless a top-down model with no guaranteed microphysical basis. In the next section, we investigate what happens if we try to reach the same point on the complexity scale by starting at the most complex point and systematically applying simplifications.

## Brownian Dynamics

The theory of MD considers systems consisting of particles, each of which exerts forces on the surrounding space, including van der Waals and electrostatic forces (Frenkel 2001; Atkins et al. 2018). Imagining a system of *N* particles, and a particle *i* with position $\mathbf {x}_i$, we can add up the forces at that point caused by all other particles in the system to get $\mathbf {F}_i(\mathbf {x}_i)$, which is the force that would be felt by particle *i*.

It is worth going into some detail about what is meant by “particle” in MD. In the most complex forms of MD, “particle” means “atom” (McCammon et al. 1977; MacKerell et al. 1998; Buch et al. 2010). Atoms which are bonded together into molecules experience spring-like forces between each other, ensuring that their average separation is equal to the empirically calculated bond length. Alternatively, at the simplest level, each molecule is considered a single particle, and so forces act on the centre of mass of the molecule rather than its constituent atoms (Alder and Wainwright 1959, 1960). Naturally, there are no spring-like forces in this description since there are no chemical bonds between particles. In between these two extremes are descriptions of intermediate complexity, where each particle represents a handful of atoms. For instance, one or more water molecules could be treated as a single particle (Wang et al. 2009; Riniker and van Gunsteren 2011), or a protein molecule could be treated as a chain of particles (up to amino acids) connected by spring-like forces (bonds) (Smith and Hall 2001; Ding et al. 2005; Tozzini 2005). In the remainder of this discussion we will, for simplicity, assume the simplest form of MD: “particle” means “molecule”. Since all forces act on molecule centres of mass, issues such as molecular shape, molecular orientation and rotation are all absorbed into the force term $\mathbf {F}_i(\mathbf {x}_i)$. For all intents and purposes, it may be simpler henceforth to think of molecules as spheres, though all our arguments apply to general molecular shapes.

It is also worth saying something initially about how chemical reactions occur in MD. Reactions are typically beyond the capabilities of MD simulators: the reasons for this are complex, but fundamentally, it is because reactions are quantum mechanical (Atkins et al. 2018), while MD uses classical mechanics. There are quantum mechanical versions of MD, but they are well beyond the scope of this article and, besides, are extremely computationally intensive (Hu and Yang 2008). We will therefore postpone discussion of chemical reactions until a later point in this section.

We will now consider what we can say about the dynamics of our system of *N* particles. By Newton’s second law, the change in momentum of particle *i* is equal to $\mathbf {F}_i(\mathbf {x}_i)$. We can therefore write:

40 $$\begin{aligned} m_i\frac{\partial ^2\mathbf {x}_i}{\partial t^2}=\mathbf {F}_i(\mathbf {x}_i), \end{aligned}$$

where $m_i$ is particle *i*’s mass. This model is perfectly good in theory, but there is an issue of scale. A system of *N* particles consists of *N* coupled versions of Eq. (40), each of which is really 3 equations (assuming a three-dimensional system volume), so we end up with 3*N* coupled equations. In the vast majority of systems of interest to biologists, water molecules are by far the most numerous particles: a simple calculation shows that a typical 1$\upmu$m$^3$*Eschericia coli* cell contains upwards of $10^{10}$ molecules of water alone. Trying to solve Eq. (40) with *N* on that kind of scale is simply impossible, even for the best computers. One solution to this is to study only a very small subvolume, much smaller than a cell, possibly containing only one protein (Klepeis et al. 2009). But unfortunately the most interesting biochemistry concerns interactions between several different biological molecules, possibly located in several different parts of the cell, and systems of this kind are beyond the capabilities of molecular dynamics.

The issue, in essence, is that in order to simulate a useful number of interesting molecules a much greater number of water molecules must also be simulated. It was realised that a way around this problem might be to not simulate the water molecules explicitly, but rather to approximate the effect of water molecules on the other molecules by adding terms to Eq. (40) (Pastor et al. 1988). This would dramatically reduce *N*, and so dramatically increase the size of systems we could plausibly simulate.

**Fig. 7:**
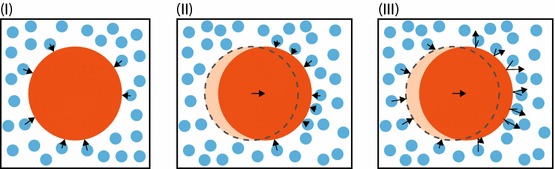
Three effects of water molecules (blue) on the motion of a large particle *X* (orange). **I** Water molecules collide on all sides of the large particle apparently at random (black arrows). If *X* is stationary, there is no directional bias to the random collisions. **II** If *X* is moving right, water molecules will collide more frequently on the particle’s right side and less frequently on its left. There will be a deterministic force opposing *X*’s velocity. **III** The arrows show how the water molecules might move in the time it would have taken them to collide with *X* had *X* been stationary (dotted circle). The water molecules on the left have not yet collided with anything, while the molecules on the right have collided and reflected away with higher speeds. They are all further to the right than they would have been otherwise, causing a net flow of water to the right

The approximation of the water molecules has three components, which have an intuitive justification. We will use the word “collide” to refer to a steric (van der Waals) interaction between particles, this can be thought of as a usual collision between hard objects (or, for simplicity, hard spheres). A graphical explanation of these effects is shown in Fig. 7

(I) When water molecules collide with a larger molecule, *X*, they will apply a force and thereby induce a change in *X*’s velocity. This is simply due to the conservation of momentum and the conservation of energy. The direction and magnitude of this change are highly variable and are well approximated by a random quantity. Because of the huge number of collisions, the central limit theorem implies that this random force will be approximately Gaussian (Loeve 1977). See Fig. 7 (I).
(II) When *X* moves with some nonzero velocity, it will tend to collide more frequently with water molecules in its direction of travel. That is, if *X* is moving to the right, it will tend to experience more collisions on its right side, and fewer on its left side, than the average predicted by (I). The number of collisions will scale with the magnitude of *X*’s velocity (since faster-moving particles will experience more such collisions in a fixed time period). See Fig. 7 (II).
(III) This component is much more subtle and takes some explaining. Let us say *X* is initially stationary and is hit on the left by a water molecule. Now *X* starts to move to the right with a fixed speed. After a short time, before it collides with anything else, it will have moved a short distance. Now we think about a potential water molecule which might be about to collide with *X*. If it is about to collide from above, below, the front or the back, then the fact that *X* has moved makes essentially no difference to its behaviour. If it is about to collide from the left, it will have to travel slightly further before it collides than it would have if *X* had not moved. If it is about to collide from the right, it will have to travel slightly less far before it collides. Now suppose the water molecule has collided, and been reflected, and is now travelling away from *X*. *On average*, the water molecule will be slightly further to the right than it would have been if *X* had been stationary. Furthermore, a post-collision water molecule moving right will be moving faster than it otherwise would have been, thanks to the extra energy from the moving *X*; similarly, a post-collision left-moving water molecule will be moving more slowly. Now imagine another large stationary molecule, *Y*, to the right of *X*. It is now slightly more likely to be hit on the left and by a faster-moving water molecule. Another large stationary molecule, *Z*, to the left of *X*, is slightly less likely to be hit on the right, and if it is hit it will likely be by a slower-moving water molecule. These two molecules will now both be slightly more likely to move right. Large molecules above, below, in front of and behind *X* will tend to be unaffected. Perhaps a more intuitive—though less microscopic—way to think of this is in terms of pressure. If *X* moves slightly to the right, it will vacate some volume to its left, so there will be a slightly lower water pressure to its left, and water molecules will rush to fill the void. It will also occupy some volume to its right, so there will be a slightly higher water pressure to its right, and water molecules will be pushed out. There will therefore be a net flow of water molecules to the right in the vicinity of *X*, and so *Y* and *Z* will tend to be moved slightly right by the flow. Mathematically, this manifests itself in a peculiar way: when effect (I) or (II) induces a force on a particle *j*, effect (III) ensures that all other particles in the system also experience a small force as a result. These forces will be separation dependent, so that two nearby particles will experience strongly correlated forces, while distant particles are weakly correlated. This effect is known as a “hydrodynamic” interaction between particles, because it is really a fluid dynamical effect (Ermak and McCammon 1978).

(I), (II) and (III) all refer to forces induced by steric interactions (collisions) between water molecules and the larger molecule *i*, but (I) is a random component, (II) is a deterministic component, and (III) is the consequence experienced by molecule *i* of components (I) and (II) acting on all other molecules in the system. The new version of Eq. (40), implementing all the components, is known as the Langevin equation, and it has the following form:

41 $$\begin{aligned} m_i\frac{\partial ^2\mathbf {x}_i}{\partial t^2}=\mathbf {F}_i(\mathbf {x}_i)+\sum _{j=1}^N\sqrt{2k_\mathrm{B}T} \left( \Gamma ^{\frac{1}{2}}\right) _{ij}\mathbf {\xi }_j-\sum _{j=1}^N \Gamma _{ij}\frac{\partial \mathbf {x}_j}{\partial t}, \end{aligned}$$

where $k_\mathrm{B}$ is the Boltzmann constant, *T* is the temperature, $\mathbf {\xi }_i$ is a standard Gaussian random vector, and $\Gamma$ is a $3N\times 3N$ matrix known as the “friction tensor”. The matrix $\Gamma$ can be split into $3\times 3$ blocks: the term $\Gamma _{ij}$ refers to the $3\times 3$ submatrix corresponding to the effect of particle *j*’s motion on particle *i*. The matrix $\Gamma ^{\frac{1}{2}}$ is the square root of the friction tensor.

Effect (I) is described by the term $\sqrt{2k_\mathrm{B}T}\left( \Gamma ^{\frac{1}{2}}\right) _{ii}\mathbf {\xi }_i$, i.e. the component of the square root friction tensor for the effect of particle *i* on itself. Similarly, effect (II) is described by $-\Gamma _{ii}\frac{\partial \mathbf {x}_i}{\partial t}$. Effect (III) is incorporated in two distinct ways: first, any random force felt by any particle *j*, $\xi _j$, has a hydrodynamic effect on particle *i*—this is the reason for the first sum in Eq. (41); secondly, the velocity of any particle *j* induces a hydrodynamic force on particle *i*—this is the reason for the second sum in Eq. (41). The hydrodynamic friction tensor $\Gamma$ mediates these two hydrodynamic contributions, and it is a complicated function of the relative positions of all the particles in the system. The details of the hydrodynamic implementations are well beyond the scope of this article; for a nice summary, see Ref. Ermak and McCammon (1978) which gives examples of the Oseen (Yamakawa 1971) and Rotne–Prager (Rotne and Prager 1969) implementations.

The hydrodynamic effect (III) is often left out of the Langevin equation, because it is subtle and complicated to implement. In that case, instead of Eq. (41) we get the much simpler form:

42 $$\begin{aligned} m_i\frac{\partial ^2\mathbf {x}_i}{\partial t^2}=\mathbf {F}_i(\mathbf {x}_i)+\sqrt{2\gamma _i k_BT}\mathbf {\xi }_i-\gamma _i\frac{\partial \mathbf {x}_i}{\partial t}, \end{aligned}$$

where $\xi _i$ is now an uncorrelated Gaussian random vector, and the scalar $\gamma _i=\Gamma _{ii}$ is the “friction coefficient”. It is easy to see the differential contributions of effects (I) and (II) in Eq. (42). However, there is no physically valid reason to eliminate the hydrodynamic effects at this stage, so we will leave it in for now and discuss when it may be appropriate to remove it later.

Equation (41) is a significant improvement on Eq. (40), and for computational simulations, it does well, but the presence of second derivatives makes it somewhat difficult to study analytically. However, a simplification can be made due to the fact that molecules in water exist in low-Reynolds number conditions, which implies that viscous effects dominate and inertial forces are negligible (Purcell 2014). The result is that $\frac{\partial ^2\mathbf {x}_i}{\partial t^2}=0$ and so Eq. (41) is simplified.

This is quite complicated, so it worth exploring what is actually happening. Let us temporarily ignore the random collisions with water molecules and think about what happens to a molecule moving through water at a constant velocity. Intuition is not very helpful here, since we are used to thinking about water from a human point of view: if a human is swimming in water at a constant speed, then stops swimming, their motion will slow until they stop completely, i.e. there is a (relatively) lengthy period of decreasing speed (acceleration). This is not what happens to a molecule (e.g. a protein) in water. A closer analogy would be a human swimming through a viscous fluid, such as treacle: when the human stops swimming, they simply stop moving instantly, there is no notable period of decreasing speed. (Of course, there is one, but it happens over such a short timescale that it is negligible.) Water from the point of view of a protein is just like this: it stops moving instantly, there is no protracted period of slowing down. This is the intuitive reason why we can set the acceleration term to zero in Eq. (41). See Ref. Purcell (2014) for an interesting discussion of this effect.

It follows that Eq. (41) becomes:

43 $$\begin{aligned} \mathbf {0}=\mathbf {F}_i(\mathbf {x}_i)+\sum _{j=1}^N \sqrt{2k_\mathrm{B}T}\left( \Gamma ^{\frac{1}{2}}\right) _{ij} \mathbf {\xi }_j-\sum _{j=1}^N \Gamma _{ij} \frac{\partial \mathbf {x}_j}{\partial t}. \end{aligned}$$

Using linear algebra techniques, this set of equations (for $i=1,\ldots ,N$) can be rearranged to give expressions for the velocity of each particle:

44 $$\begin{aligned} \frac{\partial \mathbf {x}_i}{\partial t}=\sum _{j=1}^N \left( \Gamma ^{-1}\right) _{ij}\mathbf {F}_j(\mathbf {x}_j) +\sqrt{\frac{2k_\mathrm{B}T}{\gamma _{i}}}\mathbf {\epsilon }_i, \end{aligned}$$

where $\Gamma ^{-1}$ is the matrix inverse of the hydrodynamic friction tensor $\Gamma$, and $\epsilon _i$ is a standard Gaussian random vector which (unlike $\xi _i$) is *correlated* with $\epsilon _j$ (for all $j=1,\ldots ,N$) according to a complicated hydrodynamic function (Ermak and McCammon 1978). The effect of the linear algebraic rearrangement is to replace the sum of *velocities* in Eq. (43) with a sum of *forces* in Eq. (44). The implication is that any force between two particles (such as a steric repulsion) will induce a (typically miniscule) change in the velocity of every other particle in the system, even if the force does not affect them directly.

Equation (44) is known as Brownian dynamics (Einstein 1956), and in essence, it says that the large molecule’s velocity has a deterministic component (due to the forces between each pair of large molecules) and a random component due to random collisions with the water molecules. It is tempting to think that individual collisions with the water molecules induce a random change in the large molecule’s position, and that is what Eq. (44) seems to imply, but this is not quite correct. The individual water molecules actually induce random changes in the large molecule’s *velocity*, and these changes will tend to happen extremely frequently, so in reality several water molecules contribute to each substantive change in the large molecule’s position. This may seem like a semantic distinction, but it is worth bearing in mind that each random position increment corresponds to a large number of actual collisions.

The next simplifying step we can make is to assume that $\mathbf {F}_i(\mathbf {x}_i)=\mathbf {0}$ everywhere and to decorrelate the random vectors $\epsilon _i$, which essentially amounts to ignoring steric (van der Waals), electrostatic and hydrodynamic interactions. This may seem to be a preposterous approximation to make, given that Eq. (44) is already quite simple, but there is a logic behind it. If we were trying to simulate (i.e. solve) Eq. (44) on a computer, we would most likely use an Euler scheme such as the one used in Ref. Ermak and McCammon (1978). To do this, we would pick a small time-step $\Delta t$ and apply the update rule:

45 $$\begin{aligned} \mathbf {x}(t+\Delta t)=\mathbf {x}(t)+\sum _{j=1}^N \left( \Gamma ^{-1}\right) _{ij}\mathbf {F}_j(\mathbf {x}_j)\Delta t+\sqrt{\frac{2k_\mathrm{B}T}{\gamma _{i}}}\mathbf {\epsilon }_i\Delta t \end{aligned}$$

to each particle at each time-step. This is not too computationally intensive in itself, but the problem is that for each particle the associated force function $\mathbf {F}_i(\cdot )$, the matrix $\Gamma ^{-1}$ and the size of the correlation in $\epsilon _i$ depend on the relative locations of all the other particles in the system. All the different $\mathbf {F}_i$’s, $\left( \Gamma ^{-1}\right) _{ij}$’s and correlations must be re-evaluated at every time-step, and this is what seriously slows down the computation. So it is clearly going to be useful to assume $\mathbf {F}_i(\mathbf {x}_i)=\mathbf {0}$, and the $\epsilon _i$’s are uncorrelated, but how can we justify this physically? If the concentrations of molecules are quite dilute, so that pairs of molecules rarely come close enough to interact sterically, electrostatically or hydrodynamically, then $\mathbf {F}_i(\mathbf {x}_i)$ would genuinely be equal to zero and $\epsilon _i$’s would be genuinely uncorrelated, for the vast majority of particles, the vast majority of the time.

We can then use the following equation for BD:

46 $$\begin{aligned} \mathbf {x}_i=\sqrt{2D_i}\mathbf {W}_t, \end{aligned}$$

where $\mathbf {W}_t$ is a three-dimensional Wiener process (confusingly also known as Brownian motion) and $D_i=\frac{k_BT}{\gamma _{i}}$ is the diffusion coefficient of particle *i*. The Wiener process, $\mathbf {W}_t=(W^{(1)}_t,W^{(2)}_t,W^{(3)}_t)^T,$ is a stochastic process defined by making $W^{(i)}_{t+\Delta t}$ a Normal$\left( W^{(i)}_t,\Delta t\right)$ random variable, for $i=1,2,3$. Mathematically, $\mathbf {W}_t$ is the time integral of $\xi$ (Karlin 2014).

It is worth considering the assumption of diluteness that lies behind Eq. (46). Until very recently, and to a large extent currently, diluteness was the *sine qua non* of modelling biochemistry. This was partly due to the modeller’s preference for simple models like the REs and the RDEs, which implicitly assume diluteness, but also due to cell biologists’ incomplete knowledge about the cellular environment. Then seminal work by Zimmerman and Trach (1991) and Zimmerman and Minton (1993) introduced both modellers and cell biologists to the idea of “macromolecular crowding” (Ellis 2001), an idea which has since become extremely fashionable in fields ranging from computational physics (Torquato and Stillinger 2010; Hofling and Franosch 2013) to bioengineering (Tan et al. 2013; Chapanian et al. 2014).

The basic idea is that the cell contains high concentrations of large molecules. Zimmerman estimated that up to 30% of the internal volume of an *Eschericia coli* cell could actually be occupied by large molecules rather than water (Zimmerman and Trach 1991). Under these conditions, the behaviour of an individual molecule will be seriously affected by the other large molecules in its vicinity, even if they do not interact chemically. There is an open question of how we might accurately modify Eq. (46) to account for this effect. Perhaps the obvious answer is to take a step backwards and bring the $\mathbf {F}(\cdot )$ back into the equation, and this is a common approach (McGuffee and Elcock 2010; Bruna and Chapman 2012; Smith and Grima 2017a), but then we again have the problem of computational efficiency.

There are two other modifications to Eq. (46) which are currently in common usage. The first is to replace the diffusion coefficient *D* with a modified diffusion coefficient $\tilde{D}$, typically $\tilde{D}=D(1-\alpha \phi )$, where $\phi$ is the local fraction of volume occupied by large molecules and $\alpha$ is a constant (Weissberg 1963; Blum et al. 1989; Fanelli and McKane 2010; Galanti et al. 2014; Smith et al. 2017). The rationale is that diffusing through a crowded medium might be analogous to diffusing through a viscous medium, and so reducing the diffusion coefficient accounts for an increase in viscosity. There is some computational evidence to back this up, but insufficient for the matter to be considered solved (Saxton 1994; Klann et al. 2009).

Many scientists believe the effect of crowding is more complex than a modification of the diffusion coefficient, and so the second modification is correspondingly less simple. The idea is to replace the Wiener process $\mathbf {W}_t$ with another process with non-Gaussian increments. The rationale is that the variance of a Wiener process is proportional to *t*, and some experiments appear to show that the variance of diffusion in a crowded environment is proportional more generally to $t^\alpha$, an effect known as “anomalous diffusion”. Generally, it is observed that $\alpha <1$ inside cells, which is known as “subdiffusion” (Weiss et al. 2004; Banks and Fradin 2005); however, $\alpha \approx 1$ (“diffusion”) (Dauty and Verkman 2004) and $\alpha >1$ (“superdiffusion”) (Reverey et al. 2015) have also been observed. Variants of the Wiener process for pre-specified values of $\alpha$ are commonly used in simulations (Metzler and Klafter 2000; Marquez-Lago et al. 2012), but the idea behind this is not uncontroversial and it raises questions (Dix and Verkman 2008). For example, how do we know which value of $\alpha$ to pick in any given simulation? The debate around anomalous diffusion in the cytoplasm is still very much open, and much more experimental evidence will be needed to reach a satisfactory conclusion.

The question of how to modify Eq. (46) to include hydrodynamic effects is a difficult one and has not been studied in significant detail. It is common to either just use Eq. (44) and live with the computational cost (Ermak and McCammon 1978), or else to simply pretend hydrodynamic effects do not exist (Cichocki and Hinsen 1990; Ridgway et al. 2008; McGuffee and Elcock 2010). The latter option is surprisingly popular and can have significant negative results. Hydrodynamic interactions are believed to be amongst the most important kinds of interactions between biological molecules (Ando and Skolnick 2010), more so than electrostatic effects, and so simply ignoring them cannot be wise. There is some evidence that steric effects and hydrodynamic effects could simply cancel each other out (Felderhof 1978)—so potentially including both or neither in a model might be OK, while including just one might be a bad idea—but much more evidence is needed for this to be a practical solution. Alternatively, some authors have attempted to calculate the effect of hydrodynamic interactions on diffusion coefficients (Felderhof 1978; Ohtsuki and Okano 1982) and reaction rates (Deutch and Felderhof 1973)—then it might be possible to modify them accordingly and claim that hydrodynamics have been taken into account. This is probably the most plausible approach, but again considerably more work is needed to make this method practical.

To bring the discussion back to Brownian dynamics, there is still something missing from our model Eq. (46): How do reactions happen? Reactions with 1 reactant (i.e. unbinding reactions) are easy: each molecule which can unbind has an internal clock, and the time until unbinding is generated as an exponentially distributed random time in the future—when the clock exceeds this time, the reaction occurs. There is a question of where the daughter molecules (i.e. the products of the unbinding) should be placed, and several different methods are in use. Perhaps the simplest method is to place them both at the same location, where the parent particle was before it unbound: this is unphysical but straightforward and requires no new parameters (Lipkov et al. 2011). An alternative is to propose an “unbinding distance”, $\sigma$, and place them randomly opposite each other on a sphere of diameter $\sigma$ centred around the location of the parent particle (Andrews and Bray 2004).

**Fig. 8:**
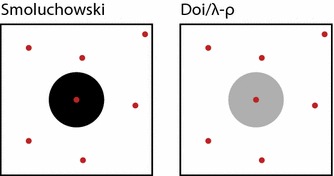
Two methods of implementing bimolecular reactions in BD. The Smoluchowski model allows particles to react immediately when the distance between the particle centres equals the sum of the particles’ reaction radii. This radius of certain interaction is shown as a solid black circle. The $\hbox {Doi}/\lambda -\rho$ model allows particles to react with probability $\lambda$ per unit time when the distance between the particle centres is less than a distance $\rho$. This radius of probabilistic interaction is shown as a solid grey circle. Note that the particle centres are denoted in red

On the other hand, modelling *bimolecular* reactions is a complicated question with a very long history. The original method, still popular today, was devised by Smoluchowski (1917), who incidentally was one of the originators of the physical theory of Brownian motion, along with Albert Einstein (Marian 1924; Einstein 1956). Smoluchowski’s idea was a simple one: each molecule is assigned a “reaction radius”, and two reacting particles will react immediately if they are brought close enough together that their reaction radii overlap (see Fig. 8). This model has advantages and disadvantages.

The advantages are that it is simple to understand and computationally and mathematically very straightforward. The most popular Brownian dynamics software, *Smoldyn* (Andrews and Bray 2004), uses the Smoluchowski model because it is so much faster than the alternatives. Mathematically, it is possible to write down the expected time until a reaction between two particles, $\mu$, as a simple function of their reaction radii ($r_1$, $r_2$), their diffusion coefficients ($D_1$, $D_2$), and the reaction volume *V*:

47 $$\begin{aligned} \mu =\frac{V}{4\pi (r_1+r_2)(D_1+D_2)}. \end{aligned}$$

Equation (47) is obtained from the diffusion equation, and a derivation can be found in Ref. Gillespie (2009a). In principle, $\mu ^{-1}$ could be put into the CME as the rate of a reaction, although this would be an approximation because the actual waiting time for Brownian collisions is not exponential (Redner 2001).

The disadvantages of Smoluchowski’s approach are slightly more complicated. Principally, the Smoluchowski model is not a particularly accurate model of how bimolecular reactions actually happen. Reacting molecules must approach each other in the correct orientation and with sufficient combined kinetic energy to exceed the activation energy of the reaction; otherwise, they will simply collide without reacting (Atkins et al. 2018). Smoluchowski does not allow for these kinds of collisions, which can occur much more frequently than successful reactions.

The second issue is a modelling issue rather than a physical one. We have already discussed the concept of “infinite diffusion”, which is unphysical but extremely useful for modelling purposes. Our ideal BD model is one where an infinite diffusion coefficient does not give nonsensical results. In the Smoluchowski model, the expected time until a reaction scales as the inverse of the diffusion coefficient, and so when diffusion is infinitely fast bimolecular reactions happen infinitely quickly. It may appear that Smoluchowski has it right here because we know from basic chemistry that if we increase the temperature of a system (and therefore the diffusion coefficients) the reactions will tend to occur more quickly (Atkins et al. 2018). But the reality is not quite so simple.

A significant source of confusion arises from the fact that a generalised bimolecular reaction rate *k* (such as the rate in the REs) really incorporates two distinct processes: a diffusive process which brings two molecules together, and a reactive process which causes them to react with some probability. Collins and Kimball go into considerable detail on this subject in Ref. Collins and Kimball (1949), as well as Gillespie later in Ref. Gillespie (2009a). They consider a physically plausible system in which hard sphere particles diffuse by Brownian motion, but over very short timescales (shorter than the time between collisions with solvent molecules) they have instantaneous random velocities given by the Maxwell–Boltzmann distribution. In this description, a reaction occurs when the instantaneous velocities of a very close pair of particles combine in such a way that they collide with an energy greater than the intrinsic activation energy of the reaction. Under these conditions, Collins and Kimball observe that the reaction rate *k* satisfies:

48 $$\begin{aligned} \frac{1}{k}=\frac{1}{k_\mathrm{d}}+\frac{1}{k_\mathrm{b}}, \end{aligned}$$

where $k_\mathrm{d}$ is the *diffusive rate* and $k_\mathrm{b}$ is the *ballistic rate*. In other words, the expected time until a reaction between a given pair of particles is the sum of the expected time until they are brought together by diffusion and the expected time until they subsequently collide with sufficient energy.

According to Collins’ and Kimball’s model (1949), the role of diffusion is to bring reactive molecules together, whereupon they can react according to some diffusion-independent process. In other words, the probability of a reaction between a sufficiently close pair of particles (in a sufficiently short time that they cannot diffuse away again) is not a function of the diffusion coefficient. When the diffusion coefficient is infinitely fast the time until a reaction, $\frac{1}{k}$, approaches $\frac{1}{k_\mathrm{b}}$ rather than 0, and so infinite diffusion seems not to pose a problem. Unfortunately, again, it is not so simple.

Since Collins’ and Kimball’s $k_\mathrm{d}$ is a linear function of the diffusion coefficient *D*, while $k_\mathrm{b}$ has no *D*-dependence, our assumption may seem to be straightforwardly valid. However, both *D* and $k_\mathrm{b}$ are functions of temperature, via the Stokes–Einstein relation and the Arrhenius equation, respectively (Atkins et al. 2018). It follows that $k_\mathrm{b}$ has an implicit *D*-dependence—e.g. if *D* is large, there is a possibility that this may be due to the temperature being large, so $k_\mathrm{b}$ may also be large. In biology (unlike chemistry) this is not a serious problem, since temperature is essentially constant for living beings. Our assumption amounts to ignoring this subtle implicit dependence, and thereby restricting our models to biological systems. We assume that it is possible to freely vary *D* without affecting the reactive component of the bimolecular reaction rate—we can think of this as varying *D* by tuning the viscosity of the solvent, which does not directly affect the reaction rate.

In this spirit, a popular alternative to the Smoluchowski model was proposed by Doi (1976), building on the work of Teramoto and Shigesada (1967). He suggests that we should associate with each pair of reacting particles a reaction rate $\lambda$ and a reaction distance $\rho$, then we assume again that waiting times between reactions are exponential, so that two particles will react in a short time-step $\Delta t$ with a probability $\lambda \Delta t$ if they are separated by a distance less than $\rho$. In other words, if two particles are sufficiently close they react with a rate $\lambda$; otherwise, they do not react (see Fig. 8). In practice, at each time-step $\Delta t$ particle positions are updated according to Eq. (45) (typically minus the force term), whereupon the distances between each pair of potentially reacting particles are calculated. For each pair, if this distance is less than the $\rho$ associated with the reaction, then the reaction occurs with probability $\lambda \Delta t$, the particles are removed from the system, and the product particles (if there are any) are put in their place.

Doi’s model, which has come to be known as the $\lambda -\rho$ model (Erban and Chapman 2009), is actually a generalisation of the Smoluchowski model, which would have $\lambda =\infty$ and $\rho$ equal to the sum of the reactants’ reaction radii (Agbanusi and Isaacson 2014). Overall, the $\lambda -\rho$ model seems to be an optimal trade-off between model simplicity and physical accuracy. The process we want to model explicitly (diffusion) is modelled explicitly, while more complex processes (reactant orientation, kinetic energy, etc.) are lumped into the rate $\lambda$. The main disadvantage is that we are assuming exponential waiting times between reactions, but this cannot really be helped without going into significantly more modelling detail and thereby incurring a significantly higher computational cost.

We have seen how the $\lambda -\rho$ model was obtained from a purely Newtonian molecular dynamics model by making a variety of assumptions and simplifications, so that unlike the RDME, $\lambda -\rho$ is a *bottom-up* model. This provides a certain guarantee about physical accuracy, and so $\lambda -\rho$ is frequently used as a “ground truth” model. However, there is still the question of how $\lambda -\rho$ fits in with the simpler models on the complexity scale: the CME, the RDEs and the REs. We therefore now analyse the limiting behaviour of $\lambda -\rho$ in the limits of fast diffusion and high concentrations

### The Limit of Fast Diffusion

To analyse the limit of fast diffusion, we assume that diffusion coefficients can be varied independently of the $\lambda$’s and $\rho$’s. We particularly focus our attention on the limiting case where all diffusion coefficients tend to infinity (the “reaction-limited” case) and particles cannot simply diffuse out of the reaction volume. (We would say that the boundaries of the volume are “reflective”.) In this case, the position of a particle at a time $t+\Delta t$ is a uniform random variable independent of its position at time *t*. To see this, note that the updated position of a particle conditioned on its previous position will be a Gaussian random variable with large variance; the boundary conditions cause this distribution to approximate a uniform distribution, and the approximation improves as the variance grows.

Now, consider a reaction of the form $A+B\rightarrow \emptyset$, with rate $\lambda$ and reaction distance $\rho$. The symbol $\emptyset$ denotes that we are not interested in the products of the reaction. The probability that a reaction occurs between a given pair of *A* and *B* molecules in a time period $[t,t+\Delta t)$ is given by:

49 $$\begin{aligned} P(\text {reaction in }[t,t+\Delta t))=K(\rho ,[t,t+\Delta t))\lambda \Delta t, \end{aligned}$$

where $K(\rho ,[t,t+\Delta t))$ represents the exact proportion of the time interval $[t,t+\Delta t)$ for which the given pairs are within a distance $\rho$ of each other. Typically, *K* not only is unknown, but also is itself a random variable, so that reactions in the $\lambda -\rho$ model are doubly stochastic processes. In the limiting case where $D \rightarrow \infty$, *K* becomes a known random variable. At any given time point in $[t,t+\Delta t)$, each molecule is uniformly distributed, so that the probability of any pair being within $\rho$ of each other is some constant *c*. The constant *c* is approximately equal to $\frac{4\pi \rho ^3}{3V}$, the proportion of the total volume occupied by a sphere of radius $\rho$; *c* is not exactly equal to this quantity because of boundary effects: if one particle is close to a boundary, there will be less space around it for a second particle to react in. However, these kinds of effects are typically numerically very small, and the approximation is usually excellent.

At any given time point in $[t,t+\Delta t)$, then, the event “*A* and *B* are within $\rho$ of each other” is governed by a Bernoulli(*c*) random variable, say *C*. If we consider *N* equally spaced time points in $[t,t+\Delta t)$, then we can approximate:

50 $$\begin{aligned} K\approx \frac{1}{\Delta t}\sum _{i=1}^NC_i\frac{\Delta t}{N}=\frac{1}{N}\sum _{i=1}^NC_i, \end{aligned}$$

where $C_i$ are independent $\hbox {Bernoulli}(c)$ random variables. The approximation converges to equality in the limit $N\rightarrow \infty$, so by the law of large numbers, we have that $K(\rho ,[t,t+\Delta t))=c$, so that

51 $$\begin{aligned} P(\text {reaction in }[t,t+\Delta t))=c\lambda \Delta t. \end{aligned}$$

This highlights the remarkable mathematical effects of allowing the limit $D \rightarrow \infty$: an unknown random variable *K* is converted into a known constant *c*. From Eq. (51) we can derive an evolution equation for $P(n_A,n_B,t)$, the probability that there are $n_A$ molecules of *A* and $n_B$ molecules of *B* at time *t*. We choose $\Delta t$ sufficiently small that at most one reaction can happen in the time period $[t,t+\Delta t)$, and we observe that the probability of a reaction happening given that we have $n_A,n_B$ molecules of *A*, *B*, respectively, is $c\lambda \Delta t n_A n_B$ [since Eq. (51) gives the probability that any given *pair* will react, and there are $n_An_B$ pairs]. The evolution equation is:

52 $$\begin{aligned} P(n_A,n_B,t+\Delta t)= & {} P(n_A+1,n_B+1,t)(n_A+1) (n_B+1)c\lambda \Delta t\nonumber \\&+\,P(n_A,n_B,t)(1-n_An_Bc\lambda \Delta t), \end{aligned}$$

which in the limit $\Delta t \rightarrow 0$ becomes:

53 $$\begin{aligned}&\frac{\hbox {d}}{\hbox {d}t}P(n_A,n_B,t)\nonumber \\&\quad =\lambda c \left[ (n_A+1)(n_B+1) P(n_A+1,n_B+1,t)-n_A n_B P(n_A,n_B,t)\right] . \end{aligned}$$

This equation is precisely the CME for the system $A+B\xrightarrow {k}\emptyset$, where $k=\lambda c V$. The CMEs for other types of mass action reactions can be derived in an analogous way, and the same will hold true. It follows that the CME *exactly* describes the evolution of molecule numbers in $\lambda -\rho$ in the limit of fast diffusion coefficients.

The CME has also been derived by Gillespie as the exact description for a system of uniformly distributed molecules which have instantaneous velocities given by the Maxwell–Boltzmann distribution and which react if they collide with sufficient combined velocity (Gillespie 1992). There is question with this derivation as to whether it is possible for a particle to be uniformly distributed in space at all times and also to have a finite velocity and specific location. The issue is that if, say, a pair of particles collide and fail to react, then they are necessarily close together and so much more likely to react than any other pair in the next short time interval. This violates the assumption of uniform distributions and also the principle of mass action. It is our belief that the CME can be considered correct if and only if there is a genuine separation of timescales between particles’ motion (i.e. diffusion) and their reaction rates—whether we think of this as “infinite diffusion” or as “reaction-limited” dynamics is essentially a semantic question.

### The Limit of High Concentrations

**Fig. 9:**
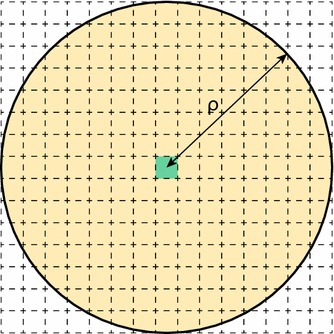
The region (orange) within a reaction distance $\rho$ of a central subvolume (green). The volume is subdivided into microscopic subvolumes, with side length substantially smaller than $\rho$

We next study the behaviour of BD when the concentration of molecules is very high. We will consider a reaction of the form $A+A\rightarrow \emptyset$ with rate $\lambda$ and reaction distance $\rho$, though the arguments can be developed analogously for other reaction types. We consider a small subvolume $\Delta V$ with a diameter substantially *smaller* than the reaction distance $\rho$. This set-up is shown in Fig. 9. A particular subvolume is shown in green, a circle with radius $\rho$ is drawn around it (orange), such that any molecule within this circle can react with any molecule in the green subvolume with rate $\lambda$. The dotted squares denote subvolumes of size $\Delta V$.

Since the concentration of molecules is very high everywhere, as long as the diffusion coefficient is nonzero, the expected concentration of molecules is locally constant inside the reaction distance and the variance is negligible. We define $\phi$ as the local expected concentration, so that the average number of molecules per subvolume is $\phi \Delta V$.

We now consider the expected change in molecule numbers in the central subvolume in a short time $\Delta t$. Temporarily ignoring diffusion, the molecule number can change for two reasons: (1) two molecules in the central subvolume (green) react with each other and (2) a molecule in the central subvolume reacts with a molecule outside the central subvolume (orange). The expected change in molecule number due to (1) is $-\,2\lambda \Delta t$ times the number of pairs in the central subvolume, i.e. $-\,2\lambda \Delta t\frac{\phi \Delta V(\phi \Delta V-1)}{2}$. Note that the initial 2 comes from the fact that a single reaction reduces the molecule number by 2. The change in molecule number due to (2) is $-\,\lambda \Delta t\phi \Delta V$ times the number of molecules outside the central subvolume but inside the reaction radius (orange), i.e. $-\,\lambda \Delta t\phi \Delta V \phi \left( V_3(\rho )-\Delta V\right)$, where $V_3(r)=\frac{4\pi r^3}{3}$ is the volume of a sphere with radius *r*. The overall expected change is then given by adding the contributions from (1) and (2):

54 $$\begin{aligned} -\,\lambda \Delta t \phi \Delta V\left[ \phi V_3(\rho )-1\right] \approx -\,\lambda V_3(\rho ) \phi ^2 \Delta V \Delta t{ ,} \end{aligned}$$

where the approximation arises from assuming the concentrations are sufficiently high that $\phi \gg \frac{1}{V_3(\rho )}$.

In the reaction–diffusion equations, the rate of change of concentrations due to the reaction $A+A\rightarrow \emptyset$ at a point *x* is given by $-2 k \phi (x)^2$, where *k* is the reaction rate and $\phi (x)$ is the local concentration near *x*. It follows that the expected change in molecule numbers in a small volume $\Delta V$ around *x* in a short time $\Delta t$ is given by:

55 $$\begin{aligned} -\,2 k \phi (x)^2\Delta V \Delta t. \end{aligned}$$

It follows that the RDEs *exactly* describe the reactive behaviour of BD with high concentrations, if we choose $k=\frac{\lambda V_3(\rho )}{2}$. The corresponding fact that the diffusion equation correctly describes Brownian diffusion is extremely well established (Gillespie and Seitaridou 2012), and we will not go into the subject here.

We note that our justification of the RDEs here implies that a molecule is unlikely to react with another molecule in the same subvolume and is much more likely to react with another molecule in a different, nearby subvolume. This makes intuitive sense when $\Delta V$ is very small, but nonetheless runs counter to the usual intuition behind the RDEs. This idea is also the basis for the derivation of the CRDME (Isaacson 2013).

**Fig. 10:**
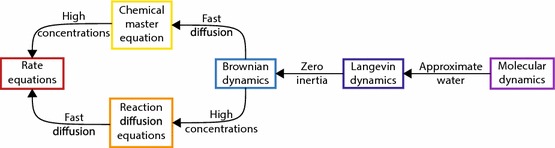
How the $\lambda -\rho$ BD model fits into the scale of model complexity shown in Fig. 1. It is the midpoint of complexity between MD and the REs

Overall, we have seen that the $\lambda -\rho$ implementation of BD is obtained from MD in a relatively rigorous manner (Erban 2014), by systematically simplifying out various effects like water and inertia. Subsequently, we found that, like the RDME, $\lambda -\rho$ can be thought of as a “parent model” of the CME, the RDEs and the REs. The relationship between all of these models is shown in Fig. 10. Just as with the RDME, if we use both $\lambda -\rho$ and the RDEs (for example) to model the same system, and the system has parameters such that we expect the RDEs to be correct, then the two models will give identical predictions. This clearly demonstrates BD’s place in the complexity scale (Fig. 1), and as a model with a sound microphysical basis and clear connections to other mainstream models, it is easy to see why BD is such a popular technique. It appears, then, that BD and the RDME have a lot in common: in the next section, we briefly assess the extent to which the two can be said to agree.

## Does the Reaction–Diffusion Master Equation Agree with Brownian Dynamics?

The $\lambda -\rho$ model has a lot in common with the RDME. Both models consider diffusing point-particles which can react with a fixed probability only if they are sufficiently close together. The rate $\lambda$ is analogous to the scaled bimolecular rate $\frac{kM}{V}$, and the subvolume size $\frac{V}{M}$ is analogous to the sphere of radius $\rho$ around the Brownian particle. The biggest difference is that the RDME discretises space, but keeps time continuous (reactions can occur at any time, but only in fixed subvolumes), whereas almost all $\lambda -\rho$ simulators discretise time and keep space continuous. (Reactions can occur anywhere, but only at time-steps of pre-specified length.) The exception is the already-mentioned CRDME (Isaacson 2013), which can be thought of as a discrete-space continuous-time $\lambda -\rho$ simulator.

It might then be surprising to learn that there is no established connection between the RDME and $\lambda -\rho$. Given a particular implementation of the $\lambda -\rho$ model for a particular system, it is typically possible to find values of *M* and reaction rates such that the RDME agrees by some metric, but if some aspect of the system (e.g. a diffusion coefficient) is changed even slightly, the optimal RDME parameters will typically have to be found again. This fact was demonstrated neatly in Ref. Smith et al. (2016). An analytical approximation to the mean molecule numbers in the RDME was obtained, which is a function of both *M* and the diffusion coefficient *D*. For any fixed choice of *M*, it was shown that this function agrees with $\lambda -\rho$ only for a specific choice of *D*. Correspondingly, for any fixed value of *D*, only a specific choice of *M* gives reasonable agreement with $\lambda -\rho$.

**Fig. 11:**
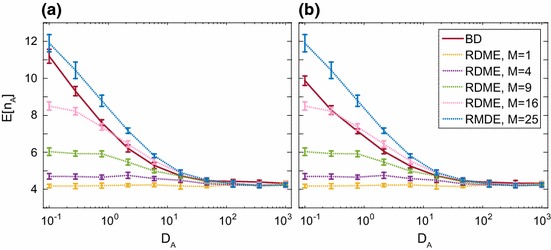
Comparison of RDME with $\lambda -\rho$ BD for the system (56), showing the dependence of the average number of *A* molecules, $\mathbb {E}[n_A]$, as a function of diffusion coefficient, $D_A$. **a** BD corresponds to $\lambda -\rho$ with spherical particles. **b** BD corresponds to $\lambda -\rho$ with cubic particles. Error bars are the standard deviation of 10 independent SSA simulations. Parameter values are $k_1=1000$, $k_2=30$, $\rho =0.1$, $\lambda =1900$, $V=1$. Simulations are performed in two spatial dimensions

To help understand this issue better, we performed simulations of the system studied in Ref. Smith et al. (2016) using both the RDME and $\lambda -\rho$. The system is straightforward and consists of a single species *A* and two reactions:

56 $$\begin{aligned} \emptyset \xrightarrow {k_1}A,~A+A\xrightarrow {k_2}\emptyset , \end{aligned}$$

where $\emptyset$ denotes that we are not interested in the species involved. The first reaction is assumed to occur at uniformly distributed points in space: it could represent transcription of a protein *A* where we do not explicitly model mRNA or ribosomes. We simulated system (56) using the RDME with a variety of values of *M* ranging from 1 to 25, and for a variety of diffusion coefficients $D_A$ ranging from $10^{-1}$ to $10^3$. In Fig. 11a we plot the mean number of *A* molecules $n_A$ as a function of $D_A$ for each choice of *M*: the results are quite remarkable.

For each value of *M* (except $M=1$) we find that the mean molecule number decreases with diffusion. When diffusion is fast, all RDME simulations agree—this should not surprise us, since we have already proved that they will all converge to the same model (the CME) in this limit. But when diffusion is slow, the RDME for each value of *M* appears to converge to a *different* limit: when $M=1$, the limit appears to be around 4, but when $M=16$ it appears to be around 9. The implication is that the RDME appears to be very sensitive to the choice of *M*, at least when diffusion is not fast (and when diffusion is fast you may as well use the CME).

Smith et al. (2016) cannot be said to be the first time an effect of this kind was noticed. It is well known (and we have already observed) that bimolecular reactions tend not to happen in the RDME when *M* is “too large”, which would naturally lead to changes in mean concentration as *M* grows (Isaacson 2013). But this is not quite the same effect as the one shown in Fig. 11a. Bimolecular reactions are happening for every choice of *M*, they are just happening at slightly different effective rates, with the result that every *M* behaves differently (not just the “too large” ones). The standard solution—to choose *M* not “too large”—does not appear to be very helpful here.

This slightly more subtle effect was observed by Erban and Chapman [who were also investigating system (56)], and they devised an ingenious solution (Erban and Chapman 2009). They suggested that the value of the bimolecular reaction rate, $k_2$, should be modified in a manner that depends on *M*, and derived a formula for this modification. If $M=1$, $k_2$ should be left as it is; if $M=4$, $k_2$ should be reduced slightly; and if $M=25$, $k_2$ should be reduced significantly. The result is a rescaled RDME whose results do not depend on *M*. In our Fig. 11a, this would lead to every RDME curve looking identical to the orange ($M=1$) curve, i.e. there is no dependence on *Mor*$D_A$. This appears to solve the problem: we can now freely vary *M* (up to a point, remember if *M* is “too large” bimolecular reactions will simply not happen) without worrying about the choice of *M* impacting on our results.

There is a possibility, however, that negates the neat solution of Erban and Chapman: What if the mean molecule numbers depend on $D_A$ for a good (i.e. physical) reason? If the choice of $D_A$ actually affects system (56) in a significant way, and we modify the RDME so that it always agrees with a model which does not depend on $D_A$ (the CME), then surely we are incorrectly modelling the system. There is a simple way to check whether the $D_A$-dependence is a real effect or an artefact of the RDME, and that is to simulate it with $\lambda -\rho$.

The solid burgundy line in Fig. 11a corresponds to an average of $\lambda -\rho$ simulations, with $\lambda$ and $\rho$ chosen to agree with the CME when $D_A$ is large. (This is always possible, as described in Sect. 3.1.) We find the same effect: the mean molecule number decreases with $D_A$, implying that this change is not an RDME artefact, but a real effect which is important to model correctly. This brings us back to our original problem: Which value of *M* should we pick? The surprising implication of Fig. 11a is that there is no definitive value of *M*. If $D_A$ is large, any value of *M* will agree with $\lambda -\rho$. In the range $10^1<D_A<10^2$, it appears that $M=9$ gives the best agreement, but for $10^0<D_A<10^1$, the choice of $M=16$ is optimal. As $D_A$ gets lower, the optimal value of *M* gets larger. This leads us to one of the main results of Smith et al. (2016): for any choice of *M* (say, $M=9$), only a subset of values of $D_A$ are accurately modelled by the RDME ($D_A>10^1$), and for any choice of $D_A$ (say, $D_A=2$), only a particular value of *M* will be optimal ($M=16$).

Optimally choosing *M* in the RDME is a difficult task, and several authors have proposed methods to solve the problem based on physical arguments (Bernstein 2005; Isaacson and Peskin 2006; Bayati et al. 2011; Kang et al. 2012). The most common approach is to choose *M* such that the diffusive timescale is substantially shorter than the reactive timescale, i.e. in a given short time-step $\Delta t$, a molecule is much more likely to diffuse into a new subvolume than it is to react (Bernstein 2005; Isaacson and Peskin 2006; Bayati et al. 2011). This will guarantee that each subvolume is well mixed, and so a CME-like description can be said to be appropriate on the subvolume scale. In the case shown in Fig. 11a, the rate of diffusion between two neighbouring subvolumes is $\frac{D_A}{h^2}$, for lattice spacing *h*; since we are considering a two-dimensional volume, we have that $h^2=\frac{V}{M}$ and that a molecule can diffuse in four different directions, so that the probability that a given molecule diffuses in $\Delta t$ is $\frac{4D_AM\Delta t}{V}$. Similarly, the rate of a bimolecular reaction in any given subvolume *i* is $\frac{k_2M}{V}n_A^{(i)}(n_A^{(i)}-1)$, where $n_A^{(i)}$ represents the number of molecules of *A* in subvolume *i*. The probability that a given molecule is involved in this bimolecular reaction in time-step $\Delta t$ is then approximately given by $\frac{k_2M}{V}\mathbb {E}[n_A^{(i)}]\Delta t$, and noting that by spatial uniformity we have $\mathbb {E}[n_A^{(i)}]=\frac{\mathbb {E}[n_A]}{M}$, we get a final probability of $\frac{k_2}{V}\mathbb {E}[n_A]\Delta t$. Overall, then, we require $M\gg \frac{k_2\mathbb {E}[n_A]}{4D_A}$. In the case where $D_A=1$, and taking $\mathbf {E}[n_A]\approx 4$ (since this is all we can say from the REs), we find the requirement $M\gg 30$, clearly at odds with Fig. 11a in which $M=16$ is optimal. However, more importantly, since Fig. 11a shows there is a single value of *M* which agrees with BD, one-sided bounds could *never* give sufficiently useful information to ensure agreement between BD and RDME. We conclude that only an estimate of the actual value of *M* (or very tight two-sided bounds on *M*) could potentially work here; however, it currently seems doubtful that such an estimate could be derived in general.

Again, we see the uneasy relationship between the RDME and $\lambda -\rho$, which both model the same kinds of systems at roughly the same level of complexity and yet never seem to agree definitively about anything. Why is this? A superficially convincing argument is to do with shape: because $\lambda -\rho$ uses the usual $L_2$ distance metric, a molecule can react with any other molecule within a spherical region around it, but the RDME typically uses cubic subvolumes, so a molecule can react only with other molecules within a cubic region. Could it be that the difference between spheres and cubes is the driver of disparity between the RDME and $\lambda -\rho$? There is a simple way to check. The $\lambda -\rho$ model can equally be defined to use other distance metrics, such as the one which leads to cubic reaction regions around molecules (Opplestrup et al. 2006). A metric of this kind was used to generate the data plotted in Fig. 11b, which compares the “cubic” $\lambda -\rho$ with the same RDME simulations as in Fig. 11a. Exactly the same effect is observed as in Fig. 11a: the optimal *M* for agreement with $\lambda -\rho$ changes as $D_A$ changes.

If it is not shape related, why then is there this discrepancy between RDME and $\lambda -\rho$? This is a relatively open question, but the most convincing argument is that modelling bimolecular reactions accurately requires fine-grained spatial resolution, something which $\lambda -\rho$ clearly has, and the RDME clearly does not. As evidence for this, consider that the CRDME is a modification of the RDME allowing it to have fine-grained spatial resolution and is proven to be able to approximate $\lambda -\rho$ to any given degree of accuracy (Isaacson 2013).

## Discussion

In this review, we have concerned ourselves with two stochastic reaction–diffusion models: the RDME and the $\lambda -\rho$ implementation of BD. These two models are very similar in complexity, and they both model the same kinds of processes at similar levels of detail. Indeed, we have seen that they both converge to the CME in the limit of fast diffusion, they both converge to the RDEs in the limit of high concentrations, and they both converge to the REs when both of these limits are applied simultaneously. And yet despite this, there appears to be no direct relationship between the RDME and $\lambda -\rho$, and they typically do not agree numerically.

Since $\lambda -\rho$ is derived in a semi-rigorous manner from an even more fundamental model, it may appear that there is no reason to use the RDME at all and that when $\lambda -\rho$ and RDME disagree, it is because the RDME is simply wrong. This is not quite correct. As we have seen, $\lambda -\rho$ and the RDME were developed to model the same kinds of systems, but they have completely different historical origins. The RDME is obtained by starting from a simple model (the REs) and working backwards, adding in stochasticity and diffusion—it is therefore a top-down model. $\lambda -\rho$ is obtained by starting from a complex model (MD) and working forwards, removing water molecules, and steric, electrostatic and hydrodynamic interactions—it is a bottom-up model. Remnants of these origins can still be seen in the RDME (the reaction rate *k* from the REs) and $\lambda -\rho$ (the reaction distance $\rho$, which is akin to a van der Waals radius from MD).

In an ideal world, where computers were infinitely fast, it may be tempting to use MD for all chemical kinetics problems, but this may not be wise. In many cases, particularly in biology, we do not know all the chemical species involved in a particular system, nor do we know exactly how many molecules of each species there might be, nor the details of the reactions between them. There are a lot of parameters in MD, and consequently, a lot of possible ways to get the model wrong. On the other hand, RE models are very popular, because for each reaction there is only one parameter (the reaction rate *k*), which can be relatively easily estimated from experimental data. It is hard to get the model *wrong*, and more likely the issue will be that the model does not incorporate some significant effect (e.g. diffusion). It is easy to turn a RE model into a RDME model: the only choice that needs to be made is the number of subvolumes, *M*. It is hard to turn a RE model into a $\lambda -\rho$ model, because for each bimolecular reaction there are two parameters to choose ($\lambda$ and $\rho$), and it is not clear how any particular choice of these parameters will relate to the *k*’s from the REs unless diffusion is very fast.

In many practical cases, the RDME model requires the fewest assumptions, and so will typically be the least wrong model. We have seen that the RDME can give very different numerical predictions depending on the choice of *M*, but typically all choices of *M* will agree *qualitatively*, and if numerical precision is not paramount the RDME may be the best choice. In short, a good rule of thumb is this: if you know a lot about the details of your system (e.g. molecular sizes), use $\lambda -\rho$; otherwise, use the RDME.

A further typical advantage of the RDME over BD, which we have not touched upon in this review, is computational speed (Erban et al. 2007). By virtue of its spatial discretisation, the RDME typically models fewer distinct quantities than BD and will tend to be substantially faster when molecule numbers are large and subvolumes are not too small. On the other hand, BD will perform better if RDME subvolumes contain very few molecules on average. In some cases, different models may be appropriate in different regions of the cell, and so a hybrid of BD and RDME may be the optimal model (Flegg et al. 2014). Alternatively, significant speed boosts can be achieved in BD using the Green’s function reaction dynamics (GFRD) approach which models Brownian diffusion in a statistically exact manner analogous to the SSA for the CME (van Zon and ten Wolde 2005; Takahashi et al. 2010). The rationale behind GFRD is that if a molecule is far from other molecules there is no point in simulating its entire trajectory of *positions* with a fixed small time-step, and it is more efficient to sample new *times* from the first passage time distribution of the time taken for it to reach a pre-specified displacement from its origin. The speed boost of GFRD relies on the assumption that this sampled time-step will be longer on average than the equivalent time-step in standard BD simulations, which is typically true for dilute systems but not when concentrations are high, making GFRD a poor choice for realistic whole-cell modelling. Furthermore, GFRD algorithms have only been derived for Smoluchowski reaction kinetics, and not the $\lambda -\rho$ kinetics for which we have argued in this review.

**Fig. 12:**
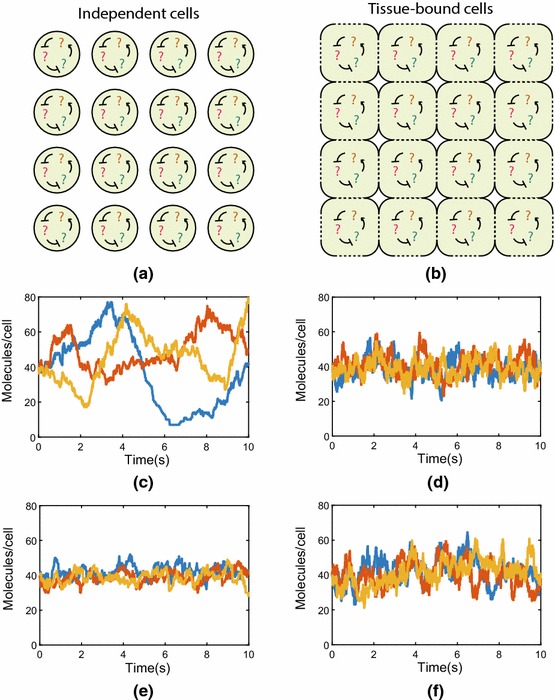
Effect of molecular transport on tissue heterogeneity. **a** A population of unconnected cells, with no intercellular transport. **b** A tissue of cells, with transport between neighbouring cells. **c** Simulations of a noisy system in unconnected cells: the cell concentrations are very heterogeneous. **d** Simulations of the same system in a connected tissue: the heterogeneity is significantly reduced. **e** Simulations of a less noisy system in unconnected cells: the cell concentrations are quite homogeneous. **f** Simulations of the same system in a connected tissue: the heterogeneity is increased

An alternative use of the RDME has recently been proposed, which eliminates the concerns about the choice of *M* (Twycross et al. 2010; Smith et al. 2016; Smith and Grima 2018). The idea is to consider each subvolume of the RDME as a cell in a tissue, and “hopping” events correspond to transport (active or passive) of molecules between adjoining cells. In this description, *M* simply refers to the number of cells in the tissue, and so is no longer an undetermined parameter. An interesting and counter-intuitive result was derived in Ref. Smith and Grima (2018) by thinking along these lines, and generalising a related result originally derived in Ref. Erdmann et al. (2009) for single-celled organisms. It was discovered that transport between cells (the analogue of diffusion in the classic RDME) has a non-trivial relationship with the heterogeneity of the tissue, i.e. the tendency for different cells in the tissue to have different concentrations. If a system has large fluctuations in concentration in a single-cell (i.e. if it is very noisy), molecular transport will tend to make the tissue more homogeneous, but if a system has small fluctuations (i.e. if it is not very noisy) then transport will tend to make the tissue more heterogeneous. This result is completely generic, though a similar result was shown by Erdmann et al. for a specific gene system. The basic principles of this are shown in Fig. 12. The idea of rationalising the RDME in this way is still in its infancy, but it is certainly promising: it could have significant usage in the modelling of cancer, which can develop stochastically in individual cells in a tissue (Gupta et al. 2011). A similar idea has also been used to model single cells by treating each physical compartment of the cell (nucleus, cytosol, etc.) as a separate subvolume of the RDME (Isaacson and Peskin 2006; Sturrock et al. 2013; Winkelmann and Schutte 2016).

We will conclude this discussion with a summary of the main open questions we have encountered in our review of the stochastic reaction–diffusion field:

- Is there a simple way to calculate RDME hopping rates for oddly shaped subvolumes?
- Can we find RDME rate functions which converge to specific non-elementary functions (e.g. Michaelis–Menten) when diffusion is fast?
- How can we modify the RDME to incorporate hydrodynamic interactions?
- How can we unify the variety of different modifications to the diffusion coefficient to account for macromolecular crowding?
- How can we incorporate hydrodynamic interactions into BD simulations without incurring a huge computational cost?
- How effective is the RDME as a model of tissue dynamics?

Answering these questions will significantly advance the field and increase our ability to model larger and larger systems in reasonable time. If we were to select a problem which, to our mind, is the most significant obstacle for the field, it would be the issue of hydrodynamic interactions. Until we find a computationally efficient method to incorporate hydrodynamic effects into BD (or RDME) simulations, the fundamental physical validity of our simulations will potentially be in doubt.

Nonetheless, no matter which model is used, the RDME and BD are guaranteed to provide insight into the link between microscopic chemistry and macroscopic biology. As it gets computationally practical to simulate systems on increasingly large scales, the RDME and BD are likely to be the models which take us beyond the state of the art in whole-cell modelling (Karr et al. 2012) to provide the first single-molecule detailed whole-cell simulations and thereby allow us to visualise the macroscopic in terms of the microscopic for the first time.
